# Molecular Determinants of Carbocation Cyclisation in Bacterial Monoterpene Synthases

**DOI:** 10.1002/cbic.202100688

**Published:** 2022-01-19

**Authors:** Nicole G. H. Leferink, Andrés M. Escorcia, Bodi R. Ouwersloot, Linus O. Johanissen, Sam Hay, Marc W. van der Kamp, Nigel S. Scrutton

**Affiliations:** ^1^ Future Biomanufacturing Research Hub Manchester Institute of Biotechnology University of Manchester 131 Princess Street Manchester M1 7DN UK; ^2^ Manchester Institute of Biotechnology and Department of Chemistry University of Manchester 131 Princess Street Manchester M1 7DN UK; ^3^ School of Biochemistry University of Bristol University Walk Bristol BS8 1TD UK; ^4^ Centre for Computational Chemistry School of Chemistry University of Bristol Cantock's Close Bristol BS8 1TS UK

**Keywords:** enzyme catalysis, molecular dynamics, protein engineering, synthetic biology, terpenoids

## Abstract

Monoterpene synthases are often promiscuous enzymes, yielding product mixtures rather than pure compounds due to the nature of the branched reaction mechanism involving reactive carbocations. Two previously identified bacterial monoterpene synthases, a linalool synthase (bLinS) and a cineole synthase (bCinS), produce nearly pure linalool and cineole from geranyl diphosphate, respectively. We used a combined experimental and computational approach to identify critical residues involved in bacterial monoterpenoid synthesis. Phe77 is essential for bCinS activity, guiding the linear carbocation intermediate towards the formation of the cyclic α‐terpinyl intermediate; removal of the aromatic ring results in variants that produce acyclic products only. Computational chemistry confirmed the importance of Phe77 in carbocation stabilisation. Phe74, Phe78 and Phe179 are involved in maintaining the active site shape in bCinS without a specific role for the aromatic ring. Phe295 in bLinS, and the equivalent Ala301 in bCinS, are essential for linalool and cineole formation, respectively. Where Phe295 places steric constraints on the carbocation intermediates, Ala301 is essential for bCinS initial cyclisation and activity. Our multidisciplinary approach gives unique insights into how carefully placed amino acid residues in the active site can direct carbocations down specific paths, by placing steric constraints or offering stabilisation via cation‐π interactions.

## Graphical abstract

**Active site residues** in terpene synthases each have a unique and precise function in guiding the reactive carbocation intermediates to the final product. Here we used a combined experimental and computational approach to unravel the role of several active site residues in bacterial monoterpene synthases. These residues are often not conserved even in closely related enzymes demonstrating the importance of a tailored active site for each terpene product.

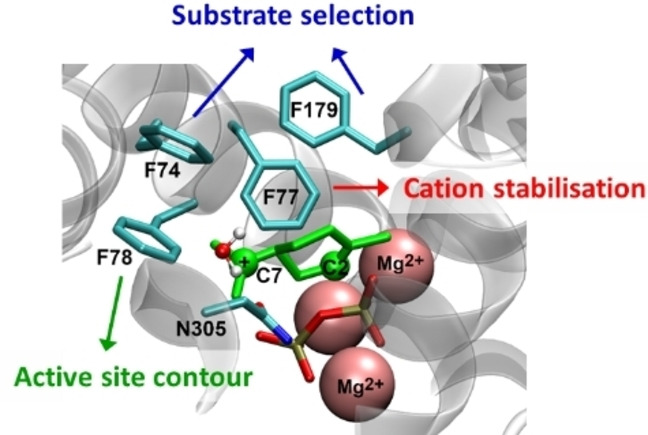

## Introduction

Monoterpenoids are industrially important natural products with applications in, for example, the flavour, fragrance, bio‐fuel and pharmaceutical industries.^[1]^ Many of the most well‐known monoterpenoids are produced by plants, where they play diverse roles in cell‐to‐cell signalling and communication, defence against predators, and attraction of pollinators.^[2]^ Recently, bacteria have also been identified as a rich source for terpene synthase activity, particularly soil‐dwelling bacteria such as *Streptomyces* species.^[3]^ Due to low terpenoid yields produced by plants and naturally occurring micro‐organisms, as well as the stereo‐chemical complexities and use of hazardous solvents for their chemical synthesis, research efforts have been directed towards the use of synthetic biology. These include the development of engineered microbes containing heterologous isoprenoid biosynthetic pathways, for the biomanufacturing of industrially important terpenoids.^[4]^

Two monoterpene synthases from the soil bacterium *Streptomyces clavuligerus* were previously identified: a bi‐functional linalool/nerolidol synthase (bLinS) and a 1,8‐cineole synthase (bCinS).^[5]^ Both enzymes were shown to outperform plant monoterpene synthases when expressed in heterologous hosts, such as *Escherichia coli*, for the biomanufacture of monoterpenoids.^[6]^ Linalool is widely used in cosmetic products such as perfumes, lotions, soaps, and shampoos, as well as in non‐cosmetic household products such as detergents, and cleaning agents. Furthermore, linalool is a vital intermediate during the manufacturing process of vitamin E.^[7]^ More recently, linalool and other mono‐ and sesquiterpenes have attracted attention as candidates for jet fuel replacements due their low freezing point and high energy density.^[8]^ Cineole (1,8‐cineole; eucalyptol) is mainly used in the flavour, fragrance, and cosmetics industries due to its pleasant minty aroma and cooling spicy taste.^[1]^

All terpenoid substrates are produced from the ubiquitous C_5_ isoprene building blocks dimethylallyl diphosphate (DMAPP) and isopentenyl diphosphate (IPP), which are delivered by the mevalonate (MVA) or non‐mevalonate biosynthetic pathways.^[9]^ Combination of DMAPP and IPP generates prenyl diphosphate substrates of varying carbon lengths, which can then be utilized by terpene synthases to produce monoterpenes (C_10_), sesquiterpenes (C_15_), diterpenes (C_20_), or larger terpene scaffolds with intricate stereo‐ and regiochemistry. Plant monoterpene synthases consist of two‐domains: a C‐terminal class I terpenoid cyclase domain and a relatively small N‐terminal domain whose function is unknown. Both bLinS and bCinS consist of a class I terpenoid cyclase domain only, and are structurally related to bacterial sesquiterpene synthases.^[6a]^ In all class I terpene synthases the reaction cascade commences by a metal‐dependent ionisation of the prenyl diphosphate substrate resulting in a highly reactive, positively charged carbocation and pyrophosphate (PPi). PPi is considered to be retained in the active site during the entire reaction cascade.^[10]^ The structural features enabling metal (magnesium) binding and ionisation are highly conserved among all class I terpene synthase enzymes, and include two metal binding motifs (the DDXXD/E motif and the DTE/NSE motif),^[11]^ and an effector triad consisting of a PPi sensor (Arg), a linker, and an effector residue.^[12]^ After ionisation, the enzyme provides a hydrophobic pocket for the carbocation to react in, where it can undergo a series of complex reactions involving rearrangements, hydride‐shifts and cyclisations, until the reaction is quenched by deprotonation or nucleophilic attack, resulting in structurally different acyclic or cyclic terpene scaffolds. The protein active site exerts both steric and electrostatic control over the cyclisation reaction cascade.^[13]^ The nature of the branched reaction mechanism, however, often results in product mixtures rather than pure products, particularly if the final product requires several re‐arrangements of the carbocation. This is undesirable in biomanufacturing processes as it reduces the overall yield and adds the need for additional purification steps of the final product. Unlike plant cineole synthases, bCinS is capable of producing nearly pure bi‐cyclic cineole from geranyl diphosphate (GPP).[^6a^, ^14^]

**Figure 1:**
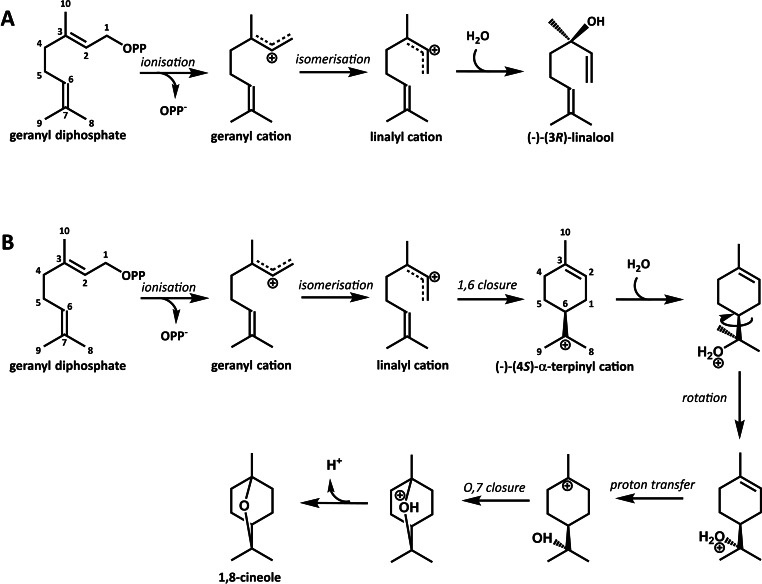
Proposed reaction cascades for the enzymatic conversion of geranyl diphosphate to (−)‐(3*R*)‐linalool by bLinS (A) and 1,8‐cineole via (−)‐(4*S*)‐α‐terpinyl by bCinS (B). The reaction catalysed by bLinS results in 100 % (−)‐(3*R*)‐linalool, and the reaction catalysed by bCinS in 96 % 1,8‐cineole, with the additional accumulation of (−)‐(4*S*)‐α‐terpineol (2 %) as well as several other products redirected from the α‐terpinyl cation, including camphene, β‐pinene and limonene (<1 % each).

bLinS and bCinS steer the initially produced geranyl cation down different stereo‐chemical paths (Figure 1).[^6a^, ^15^] In bLinS, the linalyl cation is proposed to undergo water attack to result in (−)‐(3*R*)‐linalool. In bCinS, the linalyl cation is thought to cyclises to form the (−)‐(4*S*)‐α‐terpinyl cation. Following nucleophilic water attack at position C7, a hydronium ion (R‐OH_2_^+^) is formed. After a second cyclisation followed by deprotonation, the final, achiral product 1,8‐cineole is produced.

Due to the relatively inert active sites employed by many terpene synthases to ‘manage’ the reactive carbocations, the molecular determinants for carbocation control and cyclisation are often unknown.^[13b]^ Previously, we have demonstrated the importance of Asn305 in controlling the final stages of the reaction cascade catalysed by bCinS,^[15]^ but so far little is known about carbocation control to prevent branching in the early stages of the reaction cascade leading to the cyclic α‐terpinyl intermediate.

Aromatic residues can stabilise the positive charge on carbocations via cation‐π interactions, a favoured strategy employed by terpene synthases that does not require the presence of negatively charged amino acid residues, which could result in inadvertent enzyme inactivation via active site alkylation.^[13a]^ Here we have identified a critically important phenylalanine residue involved in “gatekeeping” the cyclisation of the linalyl cation to the α‐terpinyl cation in bCinS, using a combination of experimental and computational methods. Our results indicated that the aromatic ring of Phe77 is essential for bCinS activity, guiding the carbocation intermediate, via specific stabilisation, towards the formation of the cyclic α‐terpinyl cation. Other Phe residues, including Phe74, Phe78 and Phe179 in bCinS and Phe295 in bLinS, are involved in active site contouring and restriction. By constricting the active site, substrate specificity and conformation can be carefully controlled, which is independent of side‐chain aromaticity. Our combined experimental and computational approach gives detailed mechanistic insights, which will aid the development of designer terpene synthase functionalities towards the biomanufacture of pure terpenoid products using engineered microbes.

## Results and Discussion

### Identification of target residues for mutagenesis

**Figure 2:**
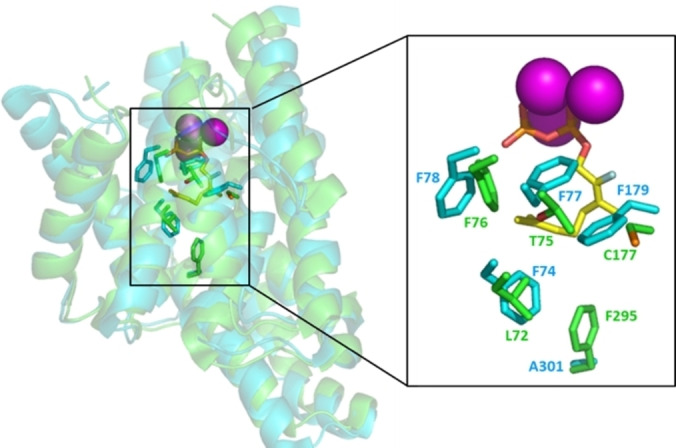
Structural overlay of linalool/nerolidol synthase (bLinS; PDB ID 5NX5) in green, and cineole synthase (bCinS; PDB ID 5NX7) in cyan from *Streptomyces clavuligerus*.^[6a]^ The fluorinated substrate analogue and Mg^2+^ ions, as bound to bCinS, are shown in yellow sticks and purple spheres respectively. Residues targeted in this study are indicated (side chain only) and shown as sticks.

Aromatic residues have been implicated in carbocation stabilisation via quadrupole mediated cation (cation‐π) interactions in many terpene synthases.^[13a]^ The crystal structures of bLinS and bCinS with bound fluorinated substrate analogues were previously solved.^[6a]^ An overlay of the bCinS and bLinS active sites reveals that bCinS contains four Phe residues close together in the active site: Phe74, Phe77, Phe78, and Phe179 (Figure 2), only one of which is conserved in bLinS (Phe76, equivalent to Phe78 in bCinS). Because bCinS does not accept the sesquiterpene precursor farnesyl diphosphate (FPP) as a substrate, but bLinS does,[^5b^, ^6a^] we assumed that at least some of these residues are likely involved in active site constriction. A multiple sequence alignment of several related bacterial class I sesquiterpene synthases for which the crystal structures are also known, revealed that many aromatic residues, including Phe, that were previously implicated in carbocation stabilisation are not generally conserved in other bacterial terpene synthases (see Figure S3 in the Supporting Information). To identify which Phe residues are important for bCinS activity, all four were subjected to Ala‐scanning mutagenesis. In addition, bLinS Phe76 (equivalent to Phe78 in bCinS) and Phe295 located at the bottom of the bLinS active site were also subjected to mutagenesis to Ala. At the same time, the equivalent residue in bCinS, Ala301, was mutated to Phe.

### Alanine scanning of active site Phe in bCinS

**Figure 3:**
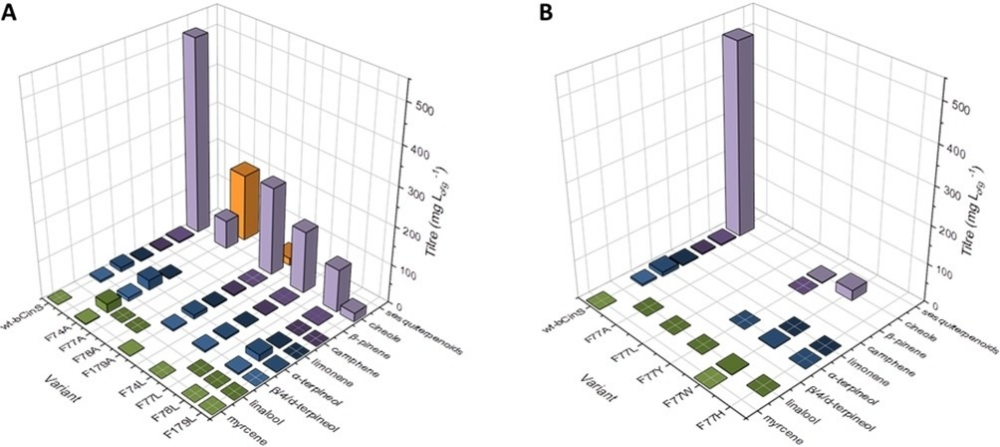
Product profiles and titres obtained for wt‐bCinS and the Phe variants upon insertion in our *E. coli* terpenoid production strain. A) Ala‐scanning of Phe 74, 77, 78 and 179 residues in the active site of bCinS, and partial product profile recovery upon insertion of the bulky, non‐aromatic hydrophobic residue Leu. B) Position 77 requires an aromatic ring for product cyclisation and cineole formation. Acyclic monoterpenoid products are shaded in green, monocyclic mono‐terpenoids in blue and bicyclic monoterpenoids in purple. Sesquiterpenoid products are shaded in orange. Product titres are calculated from 3–8 biological replicates. Geraniol, farnesol and derivatives were omitted from the comparison as they are mainly produced by endogenous *E. coli* activity.^[29]^ Sesquiterpenoid products detected are sesquicineole (71 %), α‐bisabolol (25 %), β‐sesquiphellandrene (3 %), and bisabolene (1 %) for F74A, and germacrene A (65 %), γ‐amorphene (13 %), β‐cedrene (10 %), sesquisabinene hydrate (<6 %) and β‐sesquiphellandrene (<6 %) for F179A. A full breakdown of all products detected for each variant can be found in Table S4 in the Supporting Information. Data for wt‐bCinS was obtained from Leferink et al.^[15]^

We used our previously established ‘Plug‐and‐Play’ *in vivo* terpenoid production platform in *E. coli* ^[14]^ to rapidly establish product profiles for the variant enzymes. The platform consists of an engineered *E. coli* strain containing a plasmid‐based heterologous MVA pathway (pMVA),^[16]^ and a re‐factored GPP synthase and a monoterpene synthase on a separate plasmid (pGPPSmTC/Sx). This dual‐plasmid system allows for easy switching and mutagenesis of the terpene synthases. A list of all plasmids used in this study is available online in the Supporting Information (Table S2). Due to the presence of a native FPP synthase (IspA) in our *E. coli* strain,^[17]^ the platform will also reveal any sesquiterpene synthase activity, if present. Expression of wild‐type (wt) bCinS in the terpenoid production platform results in high cineole titres (0.5 g per litre of organic overlay (L_org_^−1^)) with only minor amounts of monoterpene by‐products (2 % α‐terpineol, <1 % camphene, <1 % β‐pinene, and <1 % limonene), but no sesquiterpene products are detected (Figure 3, panel A).

Insertion of the Phe to Ala variant bCinS enzymes in the terpenoid production platform resulted in significant changes in product profiles (Figure 3, panel A). Only variants F74A and F179A appeared to exhibit activity, and in addition to the monoterpenoid products produced by wt‐bCinS, both variants also produced sesquiterpene products, with sesquicineole as the main product from F74A and germacrene A from F179A. These results reveal that the cyclisation cascade for GPP is not disrupted in either of these variants, but due to the increased active site volume, these variants can now also accept and cyclise the larger FPP substrate. This is in agreement with a recent study where the sum of the Van der Waals volumes of several non‐polar active site residues negatively correlates with the Van der Waals volumes of the preferred prenyl pyrophosphate substrates in different terpene synthase classes, such as mono‐, sesqui‐ and di‐terpene synthases.^[18]^ Also, the fact that the main sesquiterpene products accumulated by bCS‐F74A are sesquicineole and α‐bisabolol suggests that both GPP and FPP undergo the same cyclisation cascade, including Asn305 mediated water attack,^[15]^ and is further evidence that the cyclisation cascade is not disrupted by the absence of the aromatic ring at position 74. However, the sesquiterpenoid products accumulated by F179A are mostly non‐hydroxylated, indicating a difference in water reactivity for GPP and FPP in bCS‐F179A. Several minor sesquiterpenoid products were also detected for both variants, but due to the absence of authentic standards, some degree of uncertainty exists around the identification of these products due to similarities in mass spectra and retention times, and therefore retention indices, between many sesquiterpenoids (see Figure S13 and Table S3 in the Supporting Information). Variants F77A and F78A exhibited little to no activity when expressed in our engineered *E. coli* strain, with both producing minor amounts of the acyclic product linalool only, which could be the result of solvolysis of GPP in aqueous solution rather than terpene synthase activity.[^14^, ^19^]

### Phe74, Phe78 and Phe179 shape the bCinS active site

To confirm the steric effects observed in F74A and F179A, as well as to assess the importance of the aromatic ring for activity at positions 77 and 78, a bulky hydrophobic Leu residue, which lacks an aromatic ring, was introduced at all four positions. Indeed, the introduction of Leu at positions 74 and 179 resulted in product profiles that resemble that of wt‐bCinS. F74L and F179L bCinS do not produce sesquiterpene products (i. e. they do not accept FPP as substrate) and produce all/most of the monoterpenoids associated with wt‐bCinS activity (Figure 3, panel A). Introduction of Val yielded product profiles that sit in between those observed for the Ala and Leu variants (Figure S4 in the supporting information), supplying further evidence for the steric effect of residues at positions 74 and 179. A similar trend was observed for position 78, where the Ala variant did not show any activity and the introduction of increasingly larger residues yielded product profiles more like the native enzyme (Figure 3, panel A and Figure S4). These results suggest that residues Phe74, Phe78, and Phe179 are all involved in shaping and constricting the bCinS active site, with no specific role for the aromatic ring in catalysis.

### Phe77 is essential for cyclisation in bCinS

**Figure 4:**
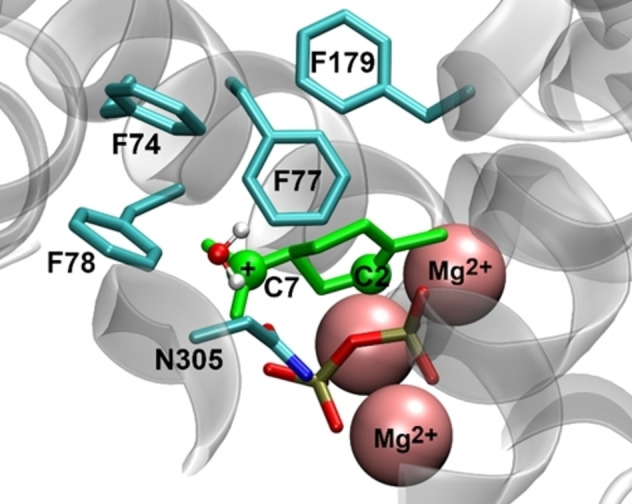
QM/MM (M06‐2X/6‐31G(d)//CHARMM36) optimized structure of the bCinS *S*‐α‐terpinyl cation complex. The cation is shown in green. Most hydrogen atoms are omitted for clarity.

For Phe77, a Leu residue could not ‘rescue’ the altered enzyme activity: both F77A and F77L mutations result in small amounts of the early exit, acyclic product linalool being produced (<0.5 mg L_org_^−1^; Figure 3, panel A). This suggests an essential role for the aromatic ring of Phe77 in carbocation stabilisation, most likely via cation‐π interactions. To further confirm this key role of Phe77, we performed multiscale (QM/MM) optimisation of the enzyme‐terpinyl cation complex (based on the structure with PDB ID 5NX7;^[6a]^ Figure 4). DFT (M06‐2X/TZVP) interaction energy calculations indicate that the presence of Phe77 stabilises the terpinyl cation significantly more than the other Phe residues (Table 1). Phe77 is closer to the main location of the positive charge in the terpinyl cation (distance of the benzyl ring centroid to C7 is 5.1 Å for Phe77 vs 5.6–6.9 Å for the other Phe residues) and is oriented more favourably for an effective cation‐π interaction than the other Phe residues (Figure 4; benzyl moiety more perpendicular to positive charge).^[20]^ Consistent with a significant cation‐π interaction for Phe77, mutation to Ala or Leu in our model significantly reduces interaction with the terpinyl cation (by 5.3 and 4.5 kcal/mol respectively; Table 1), whereas this is not the case for the other Phe residues (changes in interaction energy between −0.3 and 2.4 kcal/mol). Further, the Phe77 ring centre is located at a much shorter distance from the C2 atom of the terpinyl cation (4.7 Å vs 6.3–8.3 Å for the other residues), which suggests that it may also stabilize the preceding reaction intermediate linalyl cation (Figure 1) and the corresponding transition state connecting it to the terpinyl cation complex through cation‐π interactions. Finally, replacing Phe77 with Tyr in the optimized enzyme‐terpinyl cation complex does not reduce the interaction energy, indicating that aromaticity (to provide cation‐π interactions) at this position is key. Indeed, introduction of Trp restores cineole production, and also in F77Y, small amounts of cineole are being produced (1–2 mg L_org_^−1^). Variant F77H does not result in cineole accumulation (Figure 3, panel B), however, this variant does support a low level of cyclisation activity, with limonene and α‐terpineol as the main products, suggesting that a His residue is capable of cation stabilisation and promotion of the reaction cascade towards the α‐terpinyl intermediate. The aromatic imidazole ring of His has also been implicated in carbocation stabilisation in limonene synthase from *Mentha spicata*.^[21]^ Detailed product profiles, GCMS chromatograms, and chemical structures of products obtained for all variants created in this study are shown in the Supporting Information online (Figures S5–S13 and Tables S3–S4).

**Table 1:** Interaction energies (in kcal/mol) between the *S*‐terpinyl cation and the side chains of relevant wild‐type and variant bCinS residues, computed by QM calculations at the M06‐2X/TZVP level.

	Enzyme residue		
Residue number	PHE	ALA	LEU
74	−2.8	−0.5	−0.4
77	−5.7	−0.4	−1.2
78	−1.9	−0.1	−0.6
179	−1.2	−0.6	−1.5

The equivalent residues to Phe77 and Phe78 in bCinS appear to have similar roles in the sesquiterpene synthase pentalenene synthase from *Streptomyces exfoliatus*, where Phe76 is ideally placed to stabilise a cyclic carbocation intermediate, and Phe77 helps provide the template for the U‐shaped substrate conformation,^[22]^ with the latter Phe being strictly conserved amongst bacterial terpene synthases, including bLinS (see Figure S3). Mutagenesis confirmed the importance of Phe77 for activity in pentalenene synthase.^[11b]^ The epi‐isozizaene synthase from *Streptomyces coelicolor* also contains a tandem Phe at this position, but their functionality is different to that observed in bCinS. The aromatic ring of Phe95 in epi‐isozizaene synthase is not essential for cyclisation, but the second Phe (Phe96) seems to be critical for activity, as removal of the aromatic ring results in simple acyclic products.^[23]^ Further research revealed that the introduction of polar residues at position 96 resulted in high‐fidelity enzymes with altered product profiles, but when Phe95 was targeted the main product remained the same, confirming the role of both Phe residues in defining the active site contour.^[24]^ In selinadiene synthase from *Streptomyces pristinaespiralis* the strictly conserved Phe residue equivalent to Phe78 in bCinS was shown to be involved in carbocation stabilisation early on in the cyclisation cascade immediately following ionisation, similar to the same residue in epi‐isozizaene synthase.^[12]^ Similarly, in isoishwarane synthase from *Streptomyces lincolnensis*, the conserved Phe was found to be essential for final product formation with a critical role in enabling downstream cyclisation.^[25]^ In aristolochene synthase from *Aspergilles terreus* the Phe equivalent to Phe78 in bCinS is likely also involved in carbocation stabilisation, but here in the final stages of the reaction.^[26]^

These residues, just upstream of the strictly conserved DDXXD/E motif, are part of a previously identified plasticity region in plant monoterpene synthases.^[27]^ Although not rich in aromatic residues, changes to these residues had major impacts on activity as well as product complexity in several different plant monoterpene synthases. Phe179 in bCinS is not conserved and the aromatic ring is not required for carbocation stabilisation, although Phe198 located in a similar position in epi‐isozizaene synthase was suggested to provide carbocation stabilisation via cation‐π interactions.^[28]^

The various Phe residues in the active site of bCinS have different but precise functions. The aromatic ring of Phe77 stabilises carbocations early on in the reaction cascade, after initial formation directing the cascade towards cyclisation and formation of α‐terpinyl, the first cyclic intermediate. This is in agreement with previous research on the diterpene producing taxadiene synthase from the plant species *Taxus brevifolia*, where carbocation control via cation‐π interactions was also observed during the early stages of the reaction cascade only.^[30]^ Whereas in *A. terreus* aristolochene synthase Phe81, equivalent to Phe77 in bCinS, is likely involved in cation‐π interactions in the penultimate step of the cyclisation cascade.^[26]^ In addition, an oversized active site was implicated in product promiscuity.^[31]^ The large number of aromatic residues, including Phe74, Phe78 and Phe179, keep the bCinS active site compact and, prevent FPP from binding as well as allowing GPP to bind in a precise bend conformation, which ultimately results in a high fidelity enzyme. But an oversized active site alone is not enough to induce promiscuity, where bCS‐F74A shows demonstrably more product promiscuity, with approximately 50 % cineole originating from GPP, than wt‐bCinS, this is not the case for bCS‐F179A, which produces up to 90 % cineole from GPP. Indeed, electrostatic guidance and dynamical effects have also been implicated as major factors in steering the reaction trajectories towards product formation, and the lack of fidelity in some terpene synthases.^[10a]^

### Phe76 and Phe295 are essential for bLinS activity

**Figure 5:**
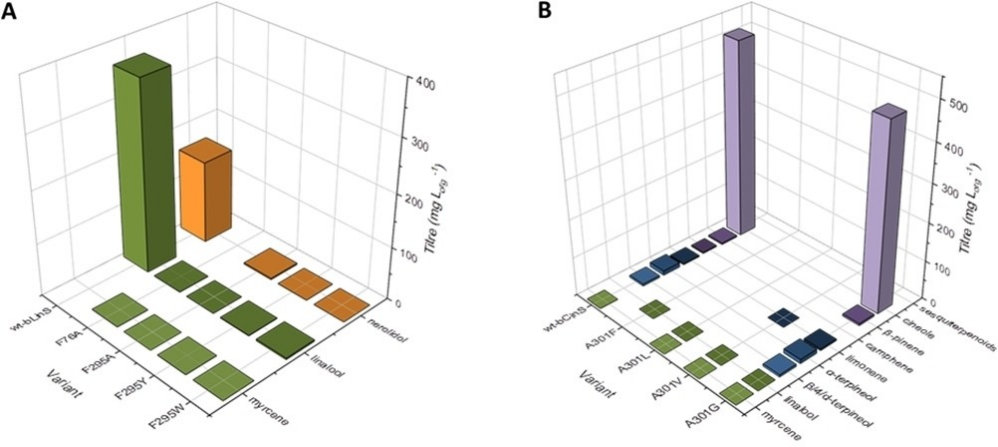
Product profiles and titres for bLinS Phe76 and Phe295 variants (A) and bCinS Ala301 variants (B) compared to the wt enzymes upon insertion in the *E. coli* terpenoid production strain. Acyclic monoterpenoid products are shaded in green, monocyclic monoterpenoids in blue and bicyclic monoterpenoids in purple. Sesquiterpenoid products are shaded in orange. Geraniol, farnesol and derivatives were omitted from the comparison as they are mainly produced by endogenous *E. coli* activity.^[29]^ A full breakdown of the product profiles can be found in Table S4 in the Supporting Information. Data for wt‐bLinS and wt‐bCinS was obtained from Leferink et al.^[15]^

Expression of wt‐bLinS, a bifunctional mono‐ and sesquiterpene synthase, results in 100 % linalool formation from GPP (0.4 g L_org_^−1^) and 100 % trans‐nerolidol from FPP (0.2 g L_org_^−1^).^[6a]^ The bLinS active site contains two Phe residues, Phe76, equivalent to Phe78 in bCinS, and Phe295. The latter is located at the bottom of the active site with the aromatic ring pointing inwards, and the equivalent residue in bCinS is Ala301 (Figure 2). Ala‐scanning of Phe76 and Phe295 resulted in variants that still produce linalool, albeit at 500–1000 fold lower product titres compared to the wt enzyme, confirming the importance of both Phe residues for efficient linalool formation (Figure 5, panel A). Interestingly, mutation of Phe76 to Ala resulted in a variant that does not produce nerolidol anymore, and mutation of Phe295 to Ala resulted in a variant that produces nerolidol as the main product. However, the aromatic rings of both Phe76 and Phe295 appear not essential for activity, and most likely have a similar role in contouring and restricting the active site as Phe74, Phe78 and Phe179 in bCinS, with Phe76 located at the same position as Phe78 in bCinS. This was further confirmed for Phe295 via introduction of Tyr and Trp at this position, two variants that were prepared previously,^[32]^ with the F295W variant producing relatively more linalool over nerolidol than wt‐bLinS. The equivalent residue to Phe295 in bLinS is conserved in selinadiene synthase from *S. pristinaespiralis*, which catalyses the formation of a bicyclic non‐hydroxylated product, where it is also believed to be involved in contouring the active site rather than carbocation stabilization.^[12]^ The total product titres of all bLinS‐Phe295 variants were over 300‐fold lower than wt‐bLinS, suggesting that the size and aromaticity of this residue is not sufficient and that the precise conformation of this residue is important for overall catalytic efficiency. Mutation of the equivalent residue in bCinS (Ala301) to Val, Leu and Phe changed the product profile significantly (Figure 5, panel B); none of the variants were able to produce cineole, with the main products being the acyclic products linalool and myrcene, and the total product titres were about 3000‐fold lower than wt‐bCinS. Only variant A301V was capable of producing very small amounts of the cyclic product limonene (0.1 mg L_org_^−1^). This suggests that the role of Ala301 is similar to Phe295 in bLinS, its precise conformation is essential for efficient formation of cineole, and likely plays an important role in the high fidelity observed for bCinS. This is in contrast to what is observed for many other monoterpene synthases, especially those from plant sources, which are generally promiscuous and show a high degree of functional plasticity.[^13b^, ^21^, ^27^, ^33^]

To understand the reason for the importance of Ala301 in forming the cyclic product cineole, we performed molecular dynamics simulations (4 independent simulations of 30 ns each) of wt‐bCinS complexed with GPP. A stable GPP conformation is observed overall, with a C1‐C6 distance around 6 Å for the majority of the time, occasionally shortening to ∼4.5 Å (suitable for cyclisation; Figure S1). In the stable bCinS‐GPP complex, Trp58 is important in forming the active site contour for the ‘cyclisation‐ready’ GPP conformation. When modelling the A301V, A301L and A301F mutations into this complex, significant clashes occur with Trp58 and other surrounding residues (Figure S2; clashes are least severe with A301V). This indicates that an active site contour supporting cyclisation cannot be maintained when introducing larger side chains than Ala at position 301. These results were further confirmed by the A301G variant, which exhibits a wt‐like product profile and titre (Figure 5, panel B).

## Conclusion

Here we have used a multidisciplinary approach to identify several important phenylalanine residues in linalool and cineole formation catalysed by two unique bacterial monoterpene synthases. All four phenylalanine residues in the bCinS active site have very specific roles. Phe74, 78 and 179 are all three involved in shaping and constricting the active site in bCinS, but where Phe74 and Phe179 prevent binding and conversion of FPP, Phe78 is important for contributing to the precise active site shape that allows cineole formation. A single mutation to Ala (F74 and F179) or Val (F74) is sufficient to convert bCinS into a bi‐functional mono‐ and sesquiterpene synthase, similar to bLinS. And whereas F78A is mostly inactive, the introduction of increasingly larger hydrophobic residues ‘restores’ the enzyme's capability for cineole formation. For all three positions the aromatic ring is not essential for activity, and mutation to bulky residues lacking an aromatic ring results in variants with product profiles that more closely resemble wt‐bCinS. In contrast, Phe77 in bCinS plays a crucial role in carbocation stabilisation early on in the reaction cascade via cation‐π interactions guiding the cation towards a single route down the cyclisation cascade, thereby preventing branching. Both Phe residues targeted in the active site of bLinS (Phe76 and Phe295) also play a crucial role in active site contouring with no specific role for the aromatic ring in carbocation stabilisation. Mutation of Ala301 in bCinS, the position equivalent to Phe295 in bLinS, indicates the importance of this position in both enzymes for final product outcome. Even though all bCinS Ala301 variants maintain some activity, any larger residue renders the enzyme incapable of producing cineole due to disruption of the active site shape.

Phenylalanine and other aromatic residues are known to play essential roles in terpene synthase catalysis, but they are often not conserved, even among closely related enzymes, demonstrating the importance of a ‘tailored’ active site for each terpene product. This emphasizes the challenge in predicting function from sequence alone in the terpene synthase protein family. Our interdisciplinary experimental and computational approach yields unique insights into how carefully placed phenylalanine residues in the active sites of terpene synthases can direct carbocations down specific paths, by placing steric constraints or offering stabilisation via cation‐π interactions, in the highly branched reaction cascade catalysed by monoterpene synthases. Such detailed understanding of the nature of the high level of fidelity demonstrated by these enzymes will aid the design of improved terpene synthase activities for biomanufacturing purposes.

## Experimental Section

**Chemicals**: All terpenoid standards used in this study, including β‐pinene, camphene, β‐myrcene, limonene, linalool, α‐terpineol, nerol, geraniol, trans‐nerolidol, and farnesol, were obtained from Sigma‐Aldrich with the exception of 1,8‐cineole, which was obtained from Tokyo Chemical Industry (TCI).

**Bacterial strains and media**: All *E. coli* strains were routinely grown in Lysogeny Broth (LB, Formedium) or on LB agar plates including antibiotic supplements as appropriate (ampicillin, 100 μg mL^−1^; kanamycin, 50 μg mL^−1^; gentamicin, 20 μg mL^−1^). For site‐directed mutagenesis, cloning, and plasmid propagation *E. coli* Stellar cells were used (Takara, Clontech). Monoterpenoid production was performed in phosphate buffered Terrific Broth (TB, Formedium) using *E. coli* DH5α cells (NEB 5α, New England Biolabs).

**Cloning and site‐directed mutagenesis**: Mutations were introduced in bCinS (WP_003952918) and bLinS (WP_003957954) using the QuikChange site‐directed mutagenesis method (Stratagene) according to the manufacturer's instructions, using plasmids pGPPSmTC/S38 and pGPPSmTC/S39 encoding N‐terminally His‐tagged native bLinS and bCinS, respectively as templates.^[6a]^ The oligonucleotides used for site‐directed mutagenesis are shown in Table S1 in the Supporting Information. Correct introduction of mutations was confirmed by standard Sanger Sequencing (Eurofins).

**Monoterpenoid production in** ***E. coli***: For monoterpenoid production, the pGPPSmTC/S plasmids were co‐transformed with plasmid pMVA into *E. coli* DH5α and grown as described.^[14]^ Briefly, expression strains were inoculated in terrific broth (TB) supplemented with 0.4 % glucose in glass screw capped vials, and induced for 48 h at 30 °C with 50 μM Isopropyl β‐D‐1‐thiogalactopyranoside (IPTG) and 25 nM anhydro‐tetracycline. A 20 % (v/v) n‐nonane layer was added to capture the volatile terpenoid products. After induction, the organic layer was collected, dried over anhydrous MgSO_4_ and mixed at a 1 : 1 ratio with ethyl acetate containing 0.01 % (v/v) sec‐butylbenzene as internal standard. The samples were analysed by GC‐MS.

**GC‐MS analysis**: Samples were injected onto an Agilent Technologies 7890B Gas Chromatograph system equipped with an Agilent Technologies 5977A MSD. The terpenoid products were separated on a DB‐WAX column (30 m×0.32 mm i.d., 0.25 μm film thickness, Agilent Technologies). The injector temperature was set at 240 °C with a split ratio of 20 : 1 (1 μL injection). The carrier gas was helium with a flow rate of 1 mL min^−1^ and a pressure of 5.1 psi. The oven program used was as follows: 50 °C (1 min hold), ramp to 68 °C at 5 °C min^−1^ (2 min hold), and ramp to 230 °C at 25 °C min^−1^ (2 min hold). The ion source temperature of the mass spectrometer (MS) was set to 230 °C and spectra were recorded from m/z 50 to m/z 250. Compound identification was carried out using authentic standards where available, or comparison to reference spectra in the NIST library of MS spectra and fragmentation patterns as described previously.^[14]^

**bCinS‐terpinyl cation interaction calculations**: Using the crystal structure of bCinS in complex with Mg^2+^ ions and the GPP analogue (2Z)‐2‐fluoro‐3,7‐dimethylocta‐2,6‐dien‐1‐yl trihydrogen diphosphate (PDB ID 5NX7^[6a]^) as starting structure, a structure of the bCinS *S*‐α‐terpinyl cation complex was generated which was optimized by quantum mechanics/molecular mechanics (QM/MM) calculation.^[34]^ The QM region (terpinyl cation, catalytic Mg^2+^ ions, and PPi) was treated at the M06‐2X/6‐31G(d) level,^[35]^ while the MM region (protein and crystal waters) was treated with the CHARMM36 force field.^[36]^ The terpinyl cation was manually docked into the enzyme active site considering the orientation/conformation of the GPP analogue in the crystal structure.[^6a^, ^15^] From the QM/MM optimized structure (Figure 4), the coordinates of the side chains of the relevant Phe residues and the terpinyl cation were extracted and the interaction energy of each Phe residue with the cation was calculated using DFT (M06‐2X/TZVP) calculations.[^35^, ^37^] Further, the side chains of the Phe residues were replaced for those of A, L and (only for Phe77) Y and the interaction energies were recalculated, for comparison. See Supporting Information for more details.

## Conflict of interest

The authors declare no conflict of interest.

1

## Supporting information

As a service to our authors and readers, this journal provides supporting information supplied by the authors. Such materials are peer reviewed and may be re‐organized for online delivery, but are not copy‐edited or typeset. Technical support issues arising from supporting information (other than missing files) should be addressed to the authors.

Supporting Information

## Data Availability

The data that support the findings of this study are available in the supplementary material of this article.
