# Supplementary material for: Molecular Determinants of Carbocation Cyclisation in Bacterial Monoterpene Synthases
Source: Chembiochem. 2022 Jan 19;23(5):e202100688. doi: 10.1002/cbic.202100688 (PMC9303655; doi:10.1002/cbic.202100688)
Supplement: Supplementary file 1 — Supporting Information [file CBIC-23-0-s001.pdf]

ChemBioChem

Supporting Information

Molecular Determinants of Carbocation Cyclisation in Bacterial Monoterpene Synthases

Nicole G. H. Leferink, Andrés M. Escorcia, Bodi R. Ouwersloot, Linus O. Johanissen, Sam Hay, Marc W. van der Kamp, and Nigel S. Scrutton*

Table of Contents

EXPERIMENTAL SECTION 2
Table S1: Oligonucleotides used in this study. 2
Table S2: Plasmids used in this study. 3
Calculation of the interaction energies between terpinyl cation and enzyme residues 4
Molecular dynamics (MD) simulations of the bCinS GPP complex 5
RESULTS SECTION 7
Figure S1: MD simulations of wt-bCinS complexed with GPP. 7
Figure S2: Modelling of the A301V/L/F mutations in the stable wt-bCinS GPP complex. 8
Figure S3: Multiple sequence alignment of bacterial terpene synthases with known structures. 9
Figure S4: Product profiles obtained for the bCinS Phe74, Phe78 and Phe179 Ala-Val-Leu variants. 10
Figure S5: GCMS analysis of terpenoid production strains containing bCinS-F74 mutants. 11
Figure S6: GCMS analysis of terpenoid production strains containing bCinS-F77 mutants. 12
Figure S7: GCMS analysis of terpenoid production strains containing bCinS-F78 mutants. 13
Figure S8: GCMS analysis of terpenoid production strains containing bCinS-F179 mutants. 14
Figure S9: GCMS analysis of terpenoid production strains containing bLinS-F76 mutants 15
Figure S10: GCMS analysis of terpenoid production strains containing bLinS-F295 mutants. 15
Figure S11: GCMS analysis of terpenoid production strains containing bCinS-A301 mutants. 16
Figure S12: Identification of monoterpenoid products in the absence of authentic standards. 17
Figure S13: Identification of sesquiterpenoids in the absence of authentic standards. 18
Table S3: Retention times and indices for all terpenoid compounds detected in this study. 19
Table S4: Product profiles and titres obtained for wt-bCinS, bLinS and all variants tested in this study. .. 20
REFERENCES 21

EXPERIMENTAL SECTION

Table S1: Oligonucleotides used in this study. Mutated codons are underlined and changed nucleotides are in lower case.

Name	Sequence (5' → 3')	Function[a]
bCinS_F74A_Fw	GGATCTGGGTTGTGATATTgcgGGTTGGTTTTTCGCCTATG	Site-directed mutagenesis
bCinS_F74V_Fw	GGATCTGGGTTGTGATATTgTgGGTTGGTTTTTCGCCTATG	Site-directed mutagenesis
bCinS_F74L_Fw	GGATCTGGGTTGTGATATTcTgGGTTGGTTTTTCGCCTATG	Site-directed mutagenesis
bCinS_F77A_Fw	GTTGTGATATTTTTGGTTGGgcgTTCGCCTATGATGATCACTTTG	Site-directed mutagenesis
bCinS_F77L_Fw	GTTGTGATATTTTTGGTTGGcTgTTCGCCTATGATGATCACTTTG	Site-directed mutagenesis
bCinS_F77Y_Fw	GTTGTGATATTTTTGGTTGGTaTTTCGCCTATGATGATCACTTTG	Site-directed mutagenesis
bCinS_F77W_Fw	GTTGTGATATTTTTGGTTGGTggTTCGCCTATGATGATCACTTTG	Site-directed mutagenesis
bCinS_F77H_Fw	GTTGTGATATTTTTGGTTGGcaTTTCGCCTATGATGATCACTTTG	Site-directed mutagenesis
bCinS_F78A_Fw	GTTGTGATATTTTTGGTTGGTTTgcCGCCTATGATGATCACTTTG	Site-directed mutagenesis
bCinS_F78V_Fw	GTGATATTTTTGGTTGGTTTgTCGCCTATGATGATCACTTTG	Site-directed mutagenesis
bCinS_F78L_Fw	GTTGTGATATTTTTGGTTGGTTTcTgGCCTATGATGATCACTTTG	Site-directed mutagenesis
bCinS_F197A_Fw	CTGCGTCATCGTACCGGTgcgATGCCTCCGCTGCTGGATCTGATTG	Site-directed mutagenesis
bCinS_F197V_Fw	CTGCGTCATCGTACCGGTgTgATGCCTCCGCTGCTGGATCTGATTG	Site-directed mutagenesis
bCinS_F197L_Fw	GTCATCGTACCGGTcTgATGCCTCCGCTGC	Site-directed mutagenesis
bCinS_A301F_Fw	GTTGAAGGTATGCGTGCAttCATTCGTGGTAATTATGATTG	Site-directed mutagenesis
bCinS_A301L_Fw	GTTGAAGGTATGCGTGCACtGATTCGTGGTAATTATGATTGG	Site-directed mutagenesis
bCinS_A301V_Fw	GTTGAAGGTATGCGTGCAGtCATTCGTGGTAATTATGATTG	Site-directed mutagenesis
bLinS_F295A_Fw	GATGCACTGAGCGCAgcgTGTCGTGGTTATCATG	Site-directed mutagenesis
bLinS_F295W_Fw	GCACTGAGCGCATggTGTCGTGGTTATC	Site-directed mutagenesis
bLinS_F295Y_Fw	GCACTGAGCGCATaTTGTCGTGGTTATC	Site-directed mutagenesis

[a]For mutagenic oligonucleotides, only the Forward primer is shown

Table S2: Plasmids used in this study.

Plasmid reference	Plasmid name	Description	Source
pMVA	pBbA5a-MTSAe-T1f-MBI(f)-T1002i	p15A, Kanr, PlacUV5, MTSA, T1, MBI-f, T1002	[1]
pGPPSmTC/S38	pBbB2a-trAgGPPS(co)- bLinS	pBBR, Ampr, Ptet, trAgGPPS(co)- bLinS	[2]
pGPPSmTC/S38-F76A	pBbB2a-trAgGPPS(co)- bLinS-F76A	pBBR, Ampr, Ptet, trAgGPPS(co)- bLinS-F76A	This study
pGPPSmTC/S38-F295A	pBbB2a-trAgGPPS(co)- bLinS-F295A	pBBR, Ampr, Ptet, trAgGPPS(co)- bLinS-F295A	This study
pGPPSmTC/S38-F295W	pBbB2a-trAgGPPS(co)- bLinS-F295W	pBBR, Ampr, Ptet, trAgGPPS(co)- bLinS-F295W	[3]
pGPPSmTC/S38-F295Y	pBbB2a-trAgGPPS(co)- bLinS-F295Y	pBBR, Ampr, Ptet, trAgGPPS(co)- bLinS-F295Y	[3]
pGPPSmTC/S39	pBbB2a-trAgGPPS(co)- bCinS	pBBR, Ampr, Ptet, trAgGPPS(co)- bCinS	[2]
pGPPSmTC/S39-F74A	pBbB2a-trAgGPPS(co)- bCinS-F74A	pBBR, Ampr, Ptet, trAgGPPS(co)- bCinS-F74A	This study
pGPPSmTC/S39-F74V	pBbB2a-trAgGPPS(co)- bCinS-F74V	pBBR, Ampr, Ptet, trAgGPPS(co)- bCinS-F74V	This study
pGPPSmTC/S39-F74L	pBbB2a-trAgGPPS(co)- bCinS-F74L	pBBR, Ampr, Ptet, trAgGPPS(co)- bCinS-F74L	This study
pGPPSmTC/S39-F77A	pBbB2a-trAgGPPS(co)- bCinS-F77A	pBBR, Ampr, Ptet, trAgGPPS(co)- bCinS-F77A	This study
pGPPSmTC/S39-F77L	pBbB2a-trAgGPPS(co)- bCinS-F77L	pBBR, Ampr, Ptet, trAgGPPS(co)- bCinS-F77L	This study
pGPPSmTC/S39-F77Y	pBbB2a-trAgGPPS(co)- bCinS-F77Y	pBBR, Ampr, Ptet, trAgGPPS(co)- bCinS-F77Y	This study
pGPPSmTC/S39-F77W	pBbB2a-trAgGPPS(co)- bCinS-F77W	pBBR, Ampr, Ptet, trAgGPPS(co)- bCinS-F77W	This study
pGPPSmTC/S39-F77H	pBbB2a-trAgGPPS(co)- bCinS-F77H	pBBR, Ampr, Ptet, trAgGPPS(co)- bCinS-F77H	This study
pGPPSmTC/S39-F78A	pBbB2a-trAgGPPS(co)- bCinS-F78A	pBBR, Ampr, Ptet, trAgGPPS(co)- bCinS-F78A	This study
pGPPSmTC/S39-F78V	pBbB2a-trAgGPPS(co)- bCinS-F78V	pBBR, Ampr, Ptet, trAgGPPS(co)- bCinS-F78V	This study
pGPPSmTC/S39-F78L	pBbB2a-trAgGPPS(co)- bCinS-F78L	pBBR, Ampr, Ptet, trAgGPPS(co)- bCinS-F78L	This study
pGPPSmTC/S39-F179A	pBbB2a-trAgGPPS(co)- bCinS-F179A	pBBR, Ampr, Ptet, trAgGPPS(co)- bCinS-F179A	This study
pGPPSmTC/S39-F179V	pBbB2a-trAgGPPS(co)- bCinS-F179V	pBBR, Ampr, Ptet, trAgGPPS(co)- bCinS-F179V	This study
pGPPSmTC/S39-F179L	pBbB2a-trAgGPPS(co)- bCinS-F179L	pBBR, Ampr, Ptet, trAgGPPS(co)- bCinS-F179L	This study
pGPPSmTC/S39-A301F	pBbB2a-trAgGPPS(co)- bCinS-A301F	pBBR, Ampr, Ptet, trAgGPPS(co)- bCinS-A301F	This study
pGPPSmTC/S39-A301L	pBbB2a-trAgGPPS(co)- bCinS-A301L	pBBR, Ampr, Ptet, trAgGPPS(co)- bCinS-A301L	This study
pGPPSmTC/S39-A301V	pBbB2a-trAgGPPS(co)- bCinS-A301V	pBBR, Ampr, Ptet, trAgGPPS(co)- bCinS-A301V	This study

Calculation of the interaction energies between terpinyl cation and enzyme residues

The crystal structure of bCinS in complex with Mg^{2+} ions and the GPP analogue (2Z)-2-fluoro-3,7-dimethylocta-2,6-dien-1-yl trihydrogen diphosphate (PDB ID 5NX7,[2] chain A) was used as starting structure to generate a structure of the bCinS *S*-α-terpinyl cation complex. The protonation states of titratable residues were estimated using PropKa3.1.[4] The aliphatic chain of the GPP analogue was removed while the phosphate motif was retained as PPi, and then the terpinyl cation was manually docked into the enzyme active site. To properly orient the terpinyl cation, we first considered the orientation/conformation of the GPP analogue in the crystal structure (this should be reasonable, considering the minimal differences in the structure of the analogue and GPP itself).[2] We further made sure that after optimization, the structure of the bCinS terpinyl cation complex corresponded to a productive configuration (i.e. it connects with the expected reactant and product complexes).

The bCinS terpinyl cation complex was optimized using quantum mechanics/molecular mechanics (QM/MM) energy minimisation,[5] where the QM region (terpinyl cation, catalytic Mg^{2+} ions, and PPi) was treated at the M06-2X/6-31G(d) level[6] and the MM region (protein and crystal waters) with the CHARMM36 force field[7] and the modified TIP3P model. The QM/MM optimizations were performed with the ChemShell package.[8] The Gaussian09[9] and DL_POLY[10] packages were used as QM and MM interfaces, respectively. All optimizations were carried out using the DL-FIND optimizer module of ChemShell and hybrid delocalized internal coordinates (HDLC).[11] The charge of the QM region was +3. All atoms within 7 Å of the QM region were unconstrained during optimizations, whereas more distant atoms were kept fixed. Non-bonded interactions were calculated without cutoff. An electrostatic embedding scheme with charge shift correction was used to compute the electrostatic interaction between the QM region and the surrounding partial charges of the MM region.[12]

From the M06-2X/6-31G(d)//CHARMM36 optimized structure (Figure 4 of the main manuscript), the coordinates of the side chains (including the alpha carbon atom) of the relevant bCinS Phe residues (74, 77, 78, and 179) and the terpinyl cation were extracted. Then the valence of alpha carbon atoms was completed by adding hydrogen atoms and the interaction energy of each Phe residue with the cation was calculated at the DFT (M06-2X/TZVP) level with counterpoise correction.[6, 13] For comparison, the Phe residues were mutated by A and L and the interaction energies were recalculated. Additionally, F77 was mutated by Y. These calculations were performed with the Gaussian09 program.[9] Mutations were performed using the mutagenesis wizard of the PyMOL package, and the mutant residues were positioned in a similar orientation to the wild type ones.

Molecular dynamics (MD) simulations of the bCinS GPP complex

We set up MD simulations of the wt-bCinS GPP complex, aiming at getting molecular insights into the effect of the A301V/L/F mutations on bCinS catalysis. The intention was to also perform simulations of the mutant bCinS GPP complexes, to assess if there were differences in the dynamics of GPP in the complex that may explain the differences in product specificity observed experimentally (similar to previous works).[14] But, as mentioned in the main manuscript and below (Figure S2), mutation of A301 into the wt-bCinS GPP complex by residues with bulkier side chains caused serious steric clashes with surrounding residues which indicate that the enzyme active-site contour promoting cyclization of GPP cannot be maintained. Due to the large uncertainty of initial structures of these mutant bCinS GPP complexes, it was therefore not possible to perform reliable simulations for these.

For the MD simulations of GPP in wt-bCinS we modelled the bCinS GPP complex again using the chain A of the 5NX7 crystal structure as starting structure.[2] The GPP fluorinated analogue was just slightly modified to meet the structure of GPP (F was removed and the aliphatic chain was protonated according to GPP). The obtained bCinS GPP structure was then solvated using a rectangular box of TIP3P water molecules with a minimum buffer of 13 Å around the protein, using the solvate plugin of the VMD package.[15] The water box contained 17646 water molecules. Additionally, with the autoionize plugin of VMD, five Na^+ ions were added to neutralize the system.[15] Thereafter, the complex was transferred to the Amber16 program (which faithfully represents CHARMM force fields) to perform MD simulations on GPUs using the PMEMD code.[16] The CHARMM36 protein force field was employed.[7a] GPP and Mg^{2+} parameters were taken from our previous works[14a, 17] and Allner et al.,[18] respectively.

Our MD protocol is analogous to previous studies[2, 14, 17, 19] and consisted of the following: i) minimization of the positions of hydrogen atoms (all heavy atoms fixed); ii) minimization of solvent (all other parts of the system fixed); iii) minimization of the entire system with positional restraints of 5 kcal mol^{-1} Å^{-2} applied to the Cα atoms and the $Mg^{2+}{}_B$ ion, and with one-sided harmonic distance restraints between diphosphate oxygens and the $Mg^{2+}{}_{A,C}$ ions (applied on distances of > 2.2 Å, with force constant 50 kcal mol^{-1} Å^{-2}, to maintain the coordination observed in X-ray crystal structures); iv) 60 ps of canonical (NVT) ensemble thermalisation to 300 K (with the positional and distance restraints still in place); v) 150 ps of isothermal-isobaric (NPT) ensemble at 300 K and 1 bar, keeping the distance restraints and gradually decreasing the restraints (from 5 to 0.5 kcal mol^{-1} Å^{-2}) on the Cα atoms and $Mg^{2+}{}_B$; vi) 30 ns NPT simulation at 300 K and 1 bar with the diphosphate-$Mg^{2+}{}_{A,C}$ distance restraints. All MD simulations were performed using periodic boundary conditions and a time step of 2 fs. A direct space cut-off of 8 Å for nonbonded interactions with PME for long-range electrostatics was used. All bonds involving hydrogen atoms were constrained by SHAKE.[20] Langevin dynamics was used for temperature control (collision frequency of 5 ps^{-1} for steps iv-v and 2 ps^{-1} for step vi), and pressure was controlled by coupling to an external bath (Amber16 default settings) for NPT conditions. A total of four independent MD simulations of 30 ns were performed by using different initial velocity distributions. The analysis of the simulations was performed using the final 20 ns of each

simulation with VMD[15] and the CPPTRAJ utility of AmberTools16.[16a] Trajectories indicated that GPP was maintained in a conformation in line with cyclisation, with a reasonably stable protein structure (Figure S1). Based on representative structures from these wt-bCinS GPP complex simulations, it is clear that the active site contour involved in maintaining the cyclisation-ready conformation will be disrupted upon mutation of A301 to amino acids with bulkier side chains, primarily due to clashes with Trp58 (Figure S2).

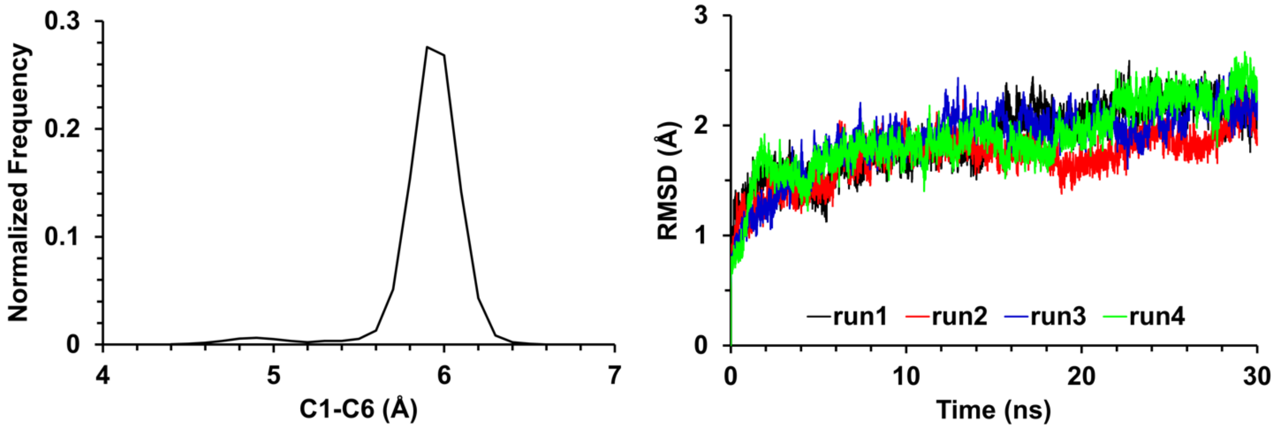

Figure S1: MD simulations of wt-bCinS complexed with GPP. Left: Histograms (in 0.1 Å bins) of the GPP:C1-C6 distance calculated over the four MD runs (80 ns in total) of the bCinS GPP complex. **Right:** Time evolution of the root mean square deviation (RMSD) for the protein backbone in the MD simulations of the bCinS GPP complex.

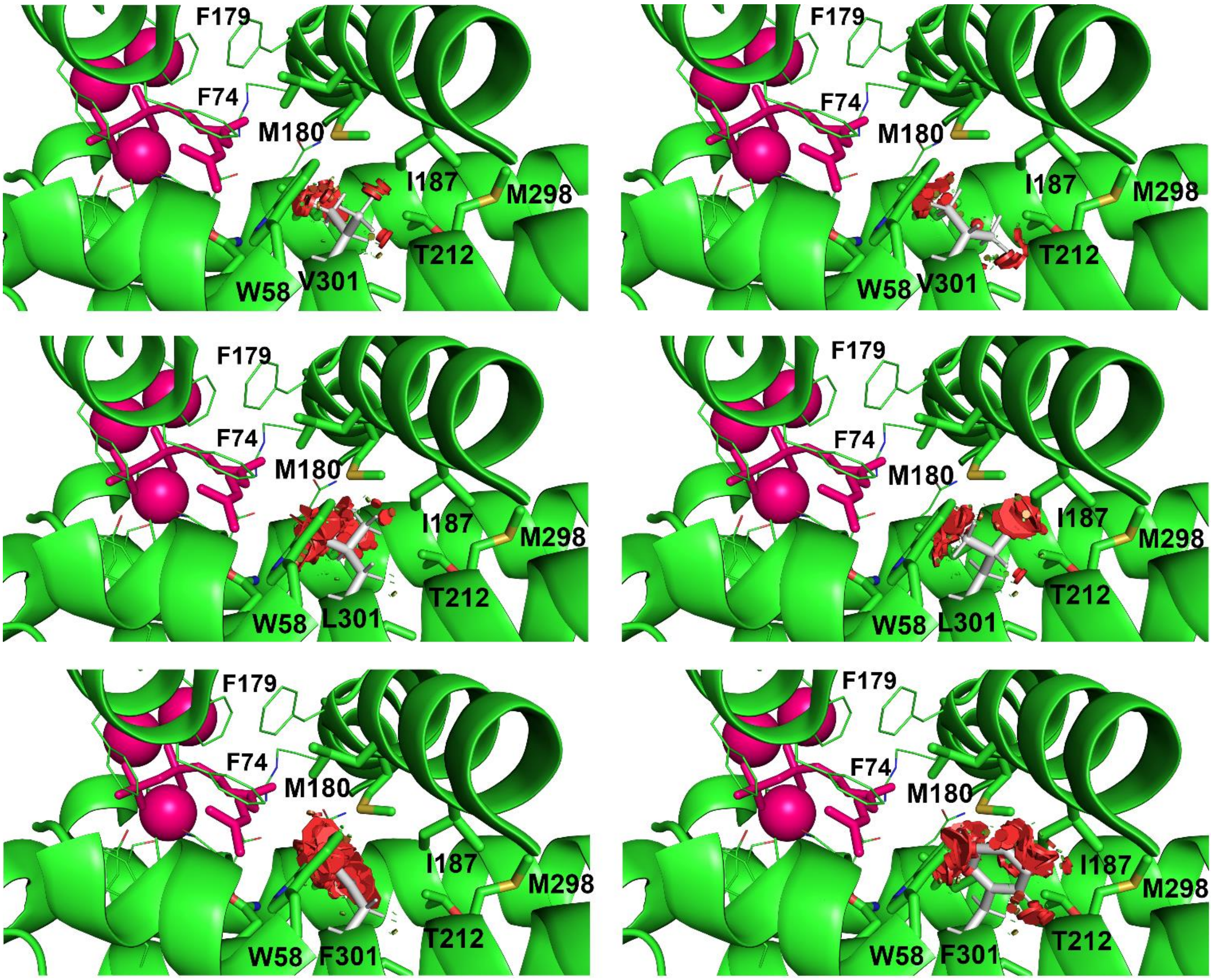

Figure S2: Modelling of the A301V/L/F mutations in the stable wt-bCinS GPP complex. Two orientations of the side chain of residue 301 (white) identified with the mutagenesis wizard of the PyMOL package for the bCinS GPP complex of the A301 mutant variants. A representative structure of the wt-bCinS GPP complex obtained from MD simulations was used as mutation target. GPP and catalytic Mg^{2+} ions are shown in magenta. Residues near residue 301 are shown in sticks. Most hydrogen atoms are omitted for clarity. Large red disks indicate significant pairwise overlap of atomic van der Waals radii, while short green lines or small green disks indicate that atoms are almost in contact or slightly overlapping. All orientations of residue 301 identified for each mutant variant showed steric contacts with surrounding residues. Similar results were obtained when using the QM/MM optimized wt-bCinS terpinyl cation complex as mutation target.

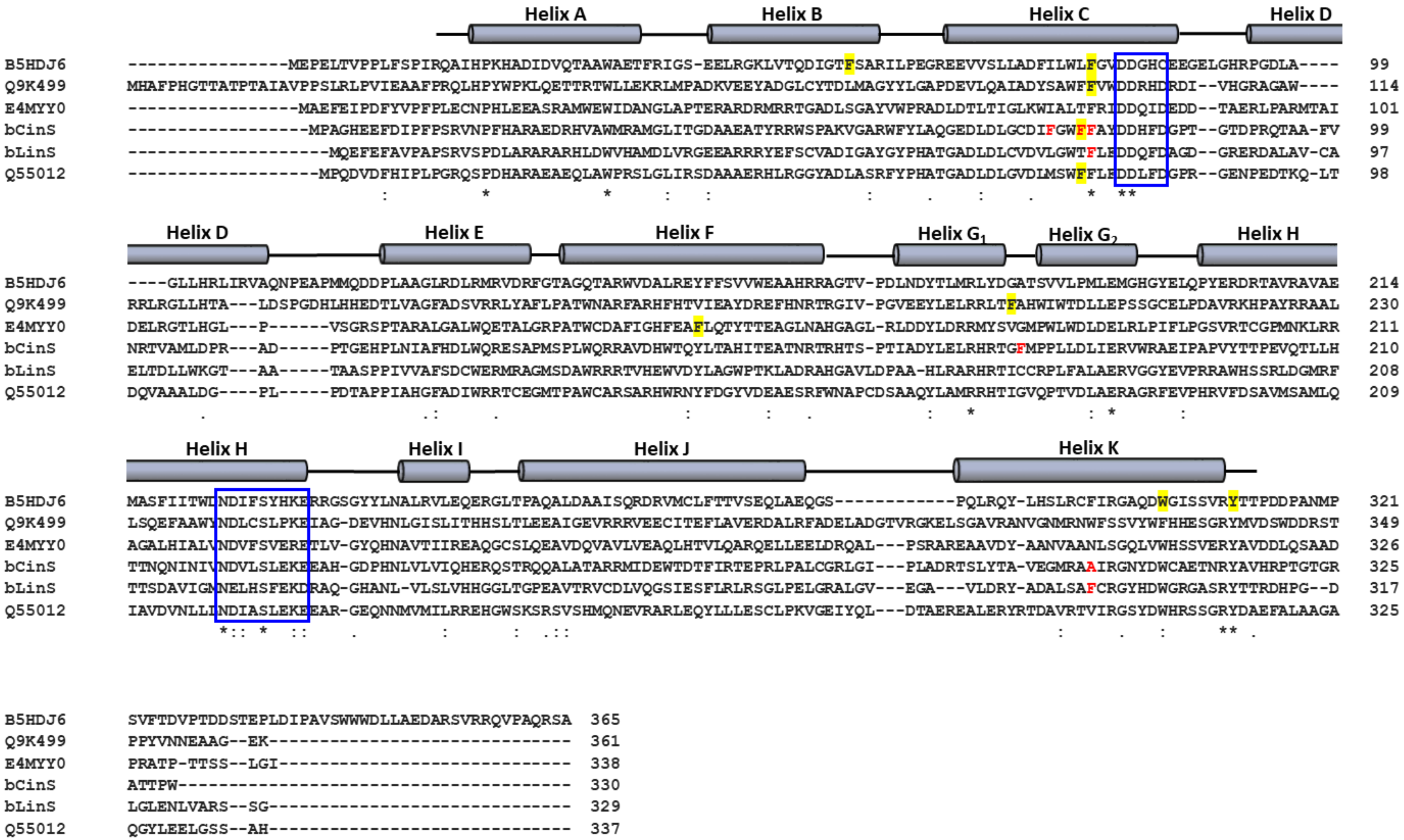

Figure S3: Multiple sequence alignment of bacterial terpene synthases with known structures. Clustal Omega multiple sequence alignment. Structural features are indicated above the alignment and the conserved DDXXD and NSE/DTE motifs are indicated with blue boxes. Aromatic residues implicated in carbocation stabilisation are highlighted in yellow. Residues mutated in this study are in bold and red. B5HDJ6 is selinadiene synthase from *Streptomyces pristinaespiralis,*[21] Q9K499 is epi-isozizaene synthase from *S. coelicolor,*[22] E4MYY0 is hedycaryol synthase from *Kitasatospora setae,*[23] and Q55012 is pentalenene synthase from *S. exfoliates*.[24]

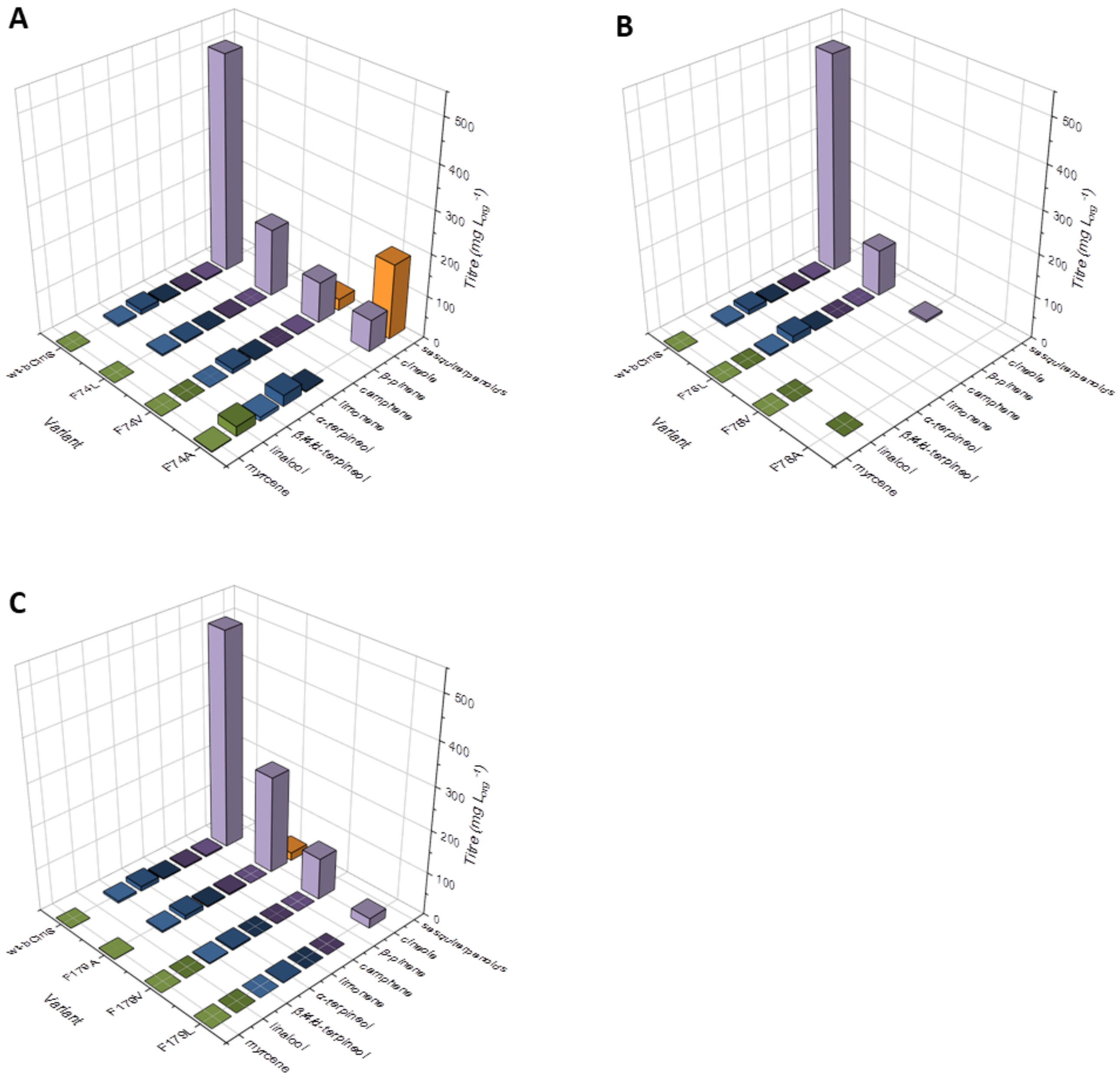

Figure S4: Product profiles obtained for the bCinS Phe74, Phe78 and Phe179 Ala-Val-Leu variants. Product profiles and titres for all assayed bCinS Phe74 (**A**), Phe78 (**B**) and Phe179 variants (**C**) compared to wt-bCinS upon insertion in the *E. coli* terpenoid production strain. Linear monoterpenoid products are shaded in green, monocyclic monoterpenoids in blue and bicyclic monoterpenoids in purple. Sesquiterpenoid products are shaded in orange. Average values of 3-8 biological replicates are shown. Geraniol, farnesol and derivatives were omitted from the comparison as they are mainly produced by endogenous *E. coli* activity.[25] Sesquiterpenoid products detected are sesquicineole (71%), α-bisabolol (25%), β-sesquiphellandrene (3%), and bisabolene (1%) for F74A, sesquicineole (47%), α-bisabolol (47%), and β-sesquiphellandrene (6%) for F74V, and germacrene A (65%), γ-amorphene (13%), β-cedrene (10%), sesquisabinene hydrate (<6%) and β-sesquiphellandrene (<6%) for F179A. A full breakdown of all products detected for each variant can be found in Table S3.

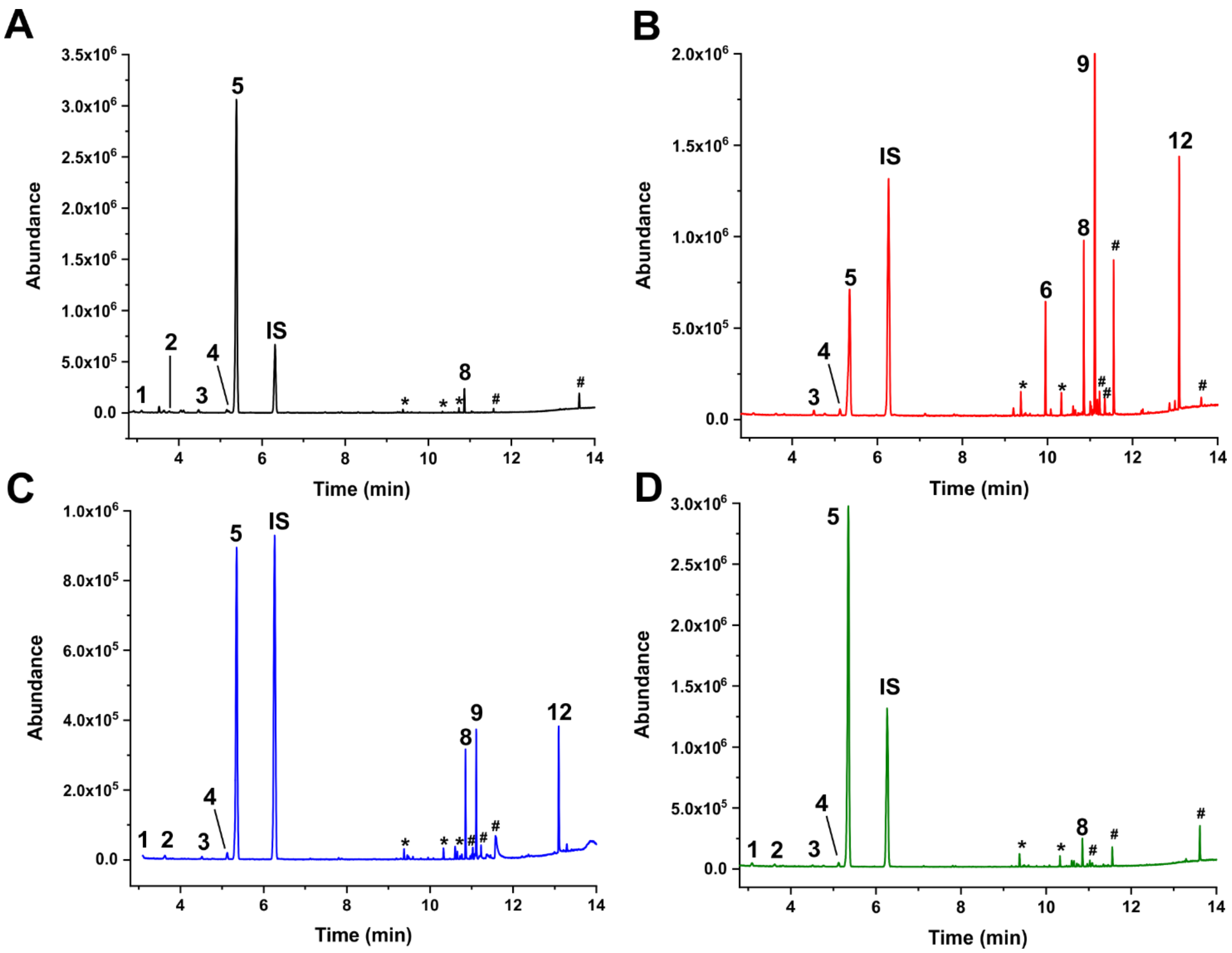

Figure S5: GCMS analysis of terpenoid production strains containing bCinS-F74 mutants. Representative total ion count chromatograms of wild-type bCinS (**A**), bCS-F74A (**B**), bCS-F74V (**C**), and bCS-F74L (**D**) from *Streptomyces clavuligerus*. Indicated terpenoid peaks are: camphene (1, rt = 3.11 min); β-pinene (2, rt = 3.65 min); β-myrcene (3, rt = 4.54 min); limonene (4, rt = 5.16 min); 1,8-cineole (5, rt = 5.39 min); linalool (6, rt = 9.98 min); γ-amorphene (7, rt = 10.78 min); α-terpineol (8, rt = 10.89 min); sesquicineole (9, rt = 11.12 min); germacrene A (10, rt = 11.18 min); nerolidol (11, rt = 12.34 min); and α-bisabolol (12, rt = 13.10 min). Minor monoterpenoid compounds detected are indicated with (*) and include β-terpineol (rt: 9.39 min), 4-terpineol (rt: 10.34 min) and δ-terpineol (rt: 10.74 min). Minor sesquiterpenoid compounds detected are indicated with (+) and include β-cedrene (rt: 10.26 min), sesquisabinene hydrate (rt: 11.09) and β-sesquiphellandrene (rt: 11.23 min). Compounds indicated with (#) are geraniol and farnesol derivatives detected due to endogenous *E. coli* activity.[25] and include neral (rt: 10.6 min), geranial (rt: 10.9 min), citronellol (rt: 11.1 min), nerol (rt: 11.3 min), geraniol (rt: 11.56), and farnesol (rt: 13.61). IS = internal standard (sec-butylbenzene).

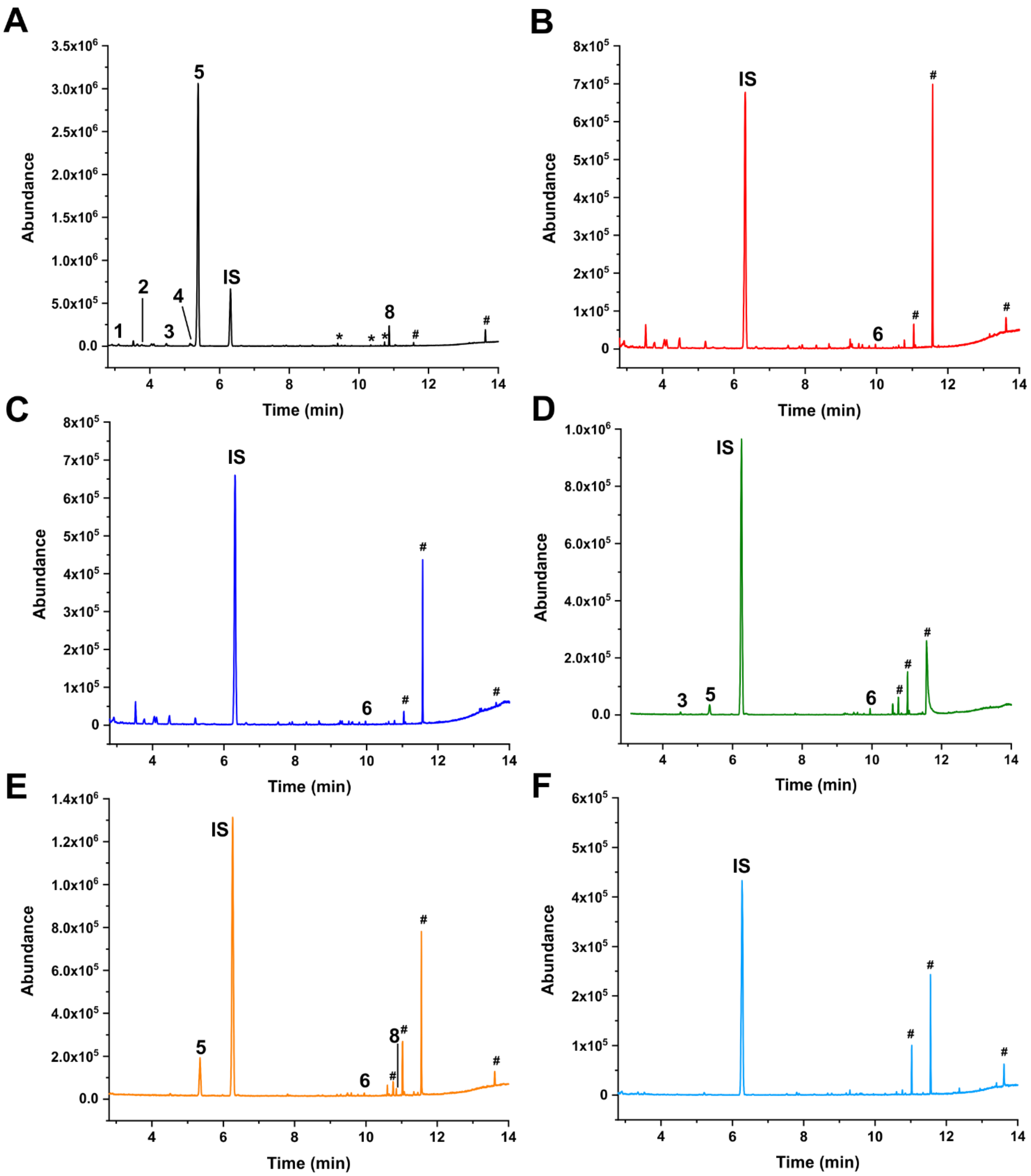

Figure S6: GCMS analysis of terpenoid production strains containing bCinS-F77 mutants. Representative total ion count chromatograms of wild-type bCinS (**A**), bCS-F77A (**B**), bCS-F77L (**C**), bCS-F77Y (**D**), bCS-F77W (**E**), and bCS-F77H (**F**) from *Streptomyces clavuligerus*. Peaks are indicated and labelled as in Figure S1.

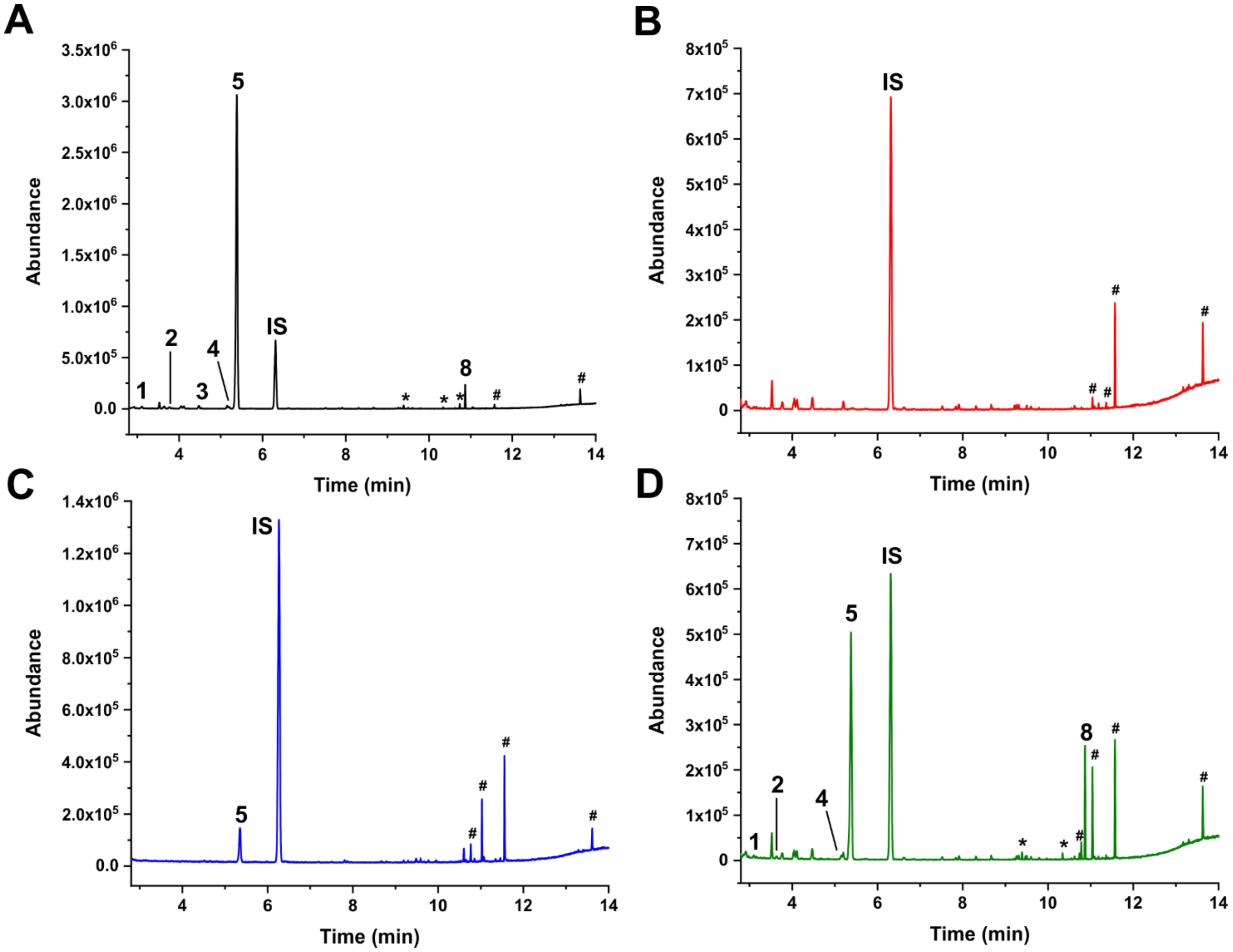

Figure S7: GCMS analysis of terpenoid production strains containing bCinS-F78 mutants. Representative total ion count chromatograms of wild-type bCinS (**A**), bCS-F78A (**B**), bCS-F78V (**C**), and bCS-F78L (**D**) from *Streptomyces clavuligerus*. Peaks are indicated and labelled as in Figure S1.

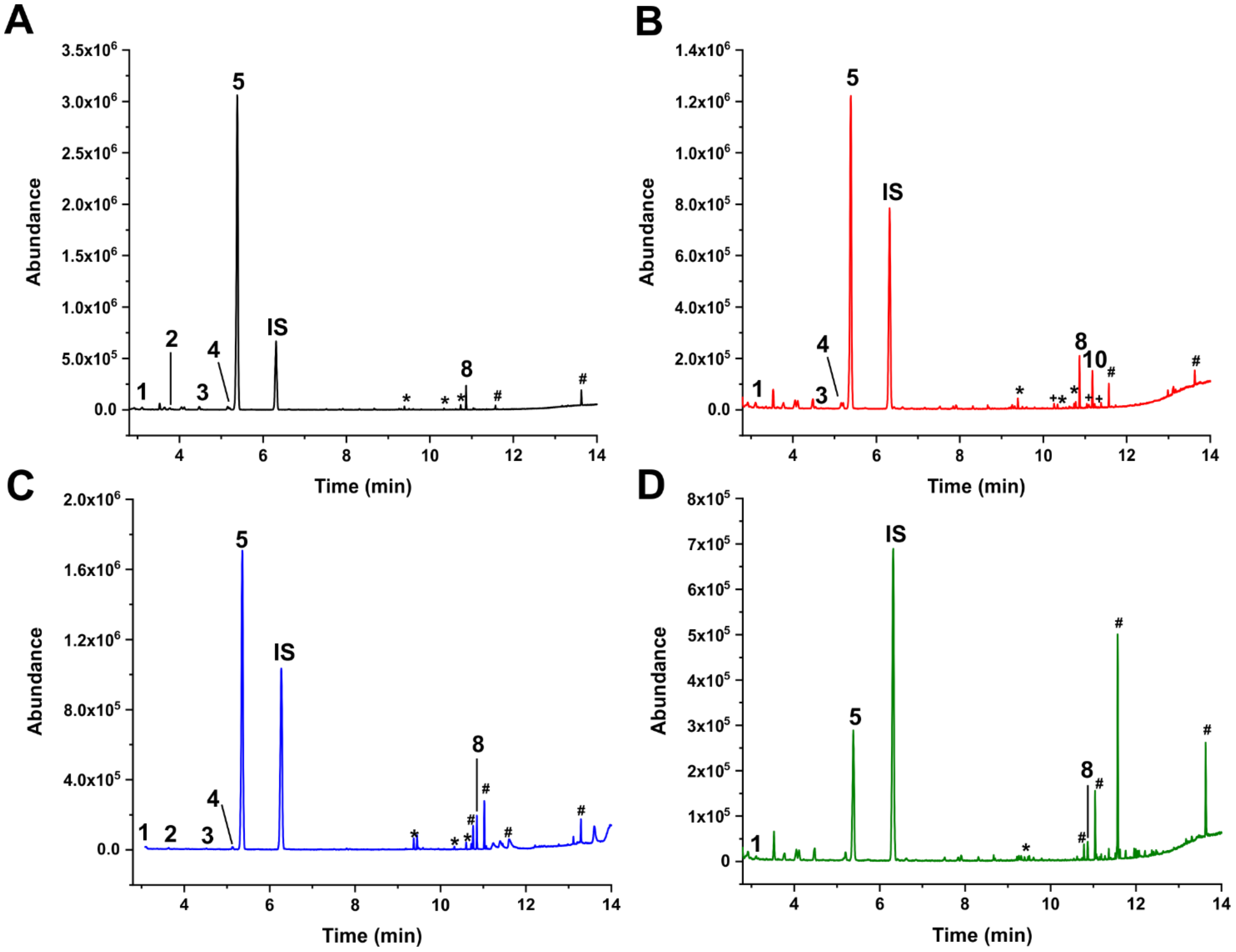

Figure S8: GCMS analysis of terpenoid production strains containing bCinS-F179 mutants. Representative total ion count chromatograms of wild-type bCinS (**A**), bCS-F179A (**B**), bCS-F179V (**C**), and bCS-F179L (**D**) from *Streptomyces clavuligerus*. Peaks are indicated and labelled as in Figure S1.

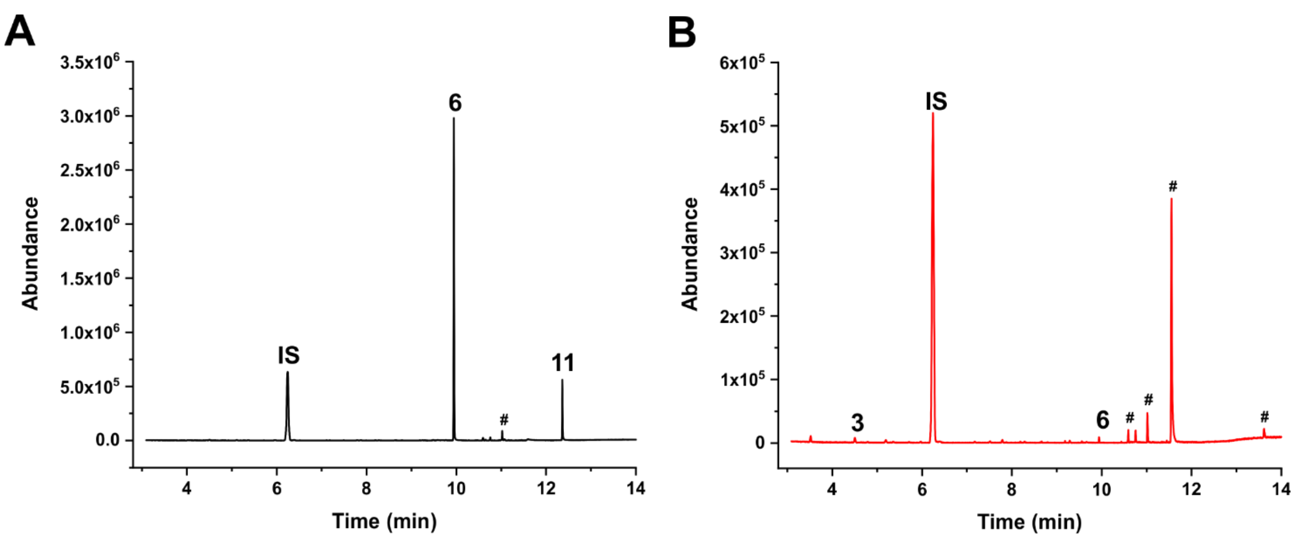

Figure S9: GCMS analysis of terpenoid production strains containing bLinS-F76 mutants. Representative total ion count chromatograms of wild-type bLinS (**A**) and bLS-F76A (**B**) from *Streptomyces clavuligerus*. Peaks are indicated and labelled as in Figure S1.

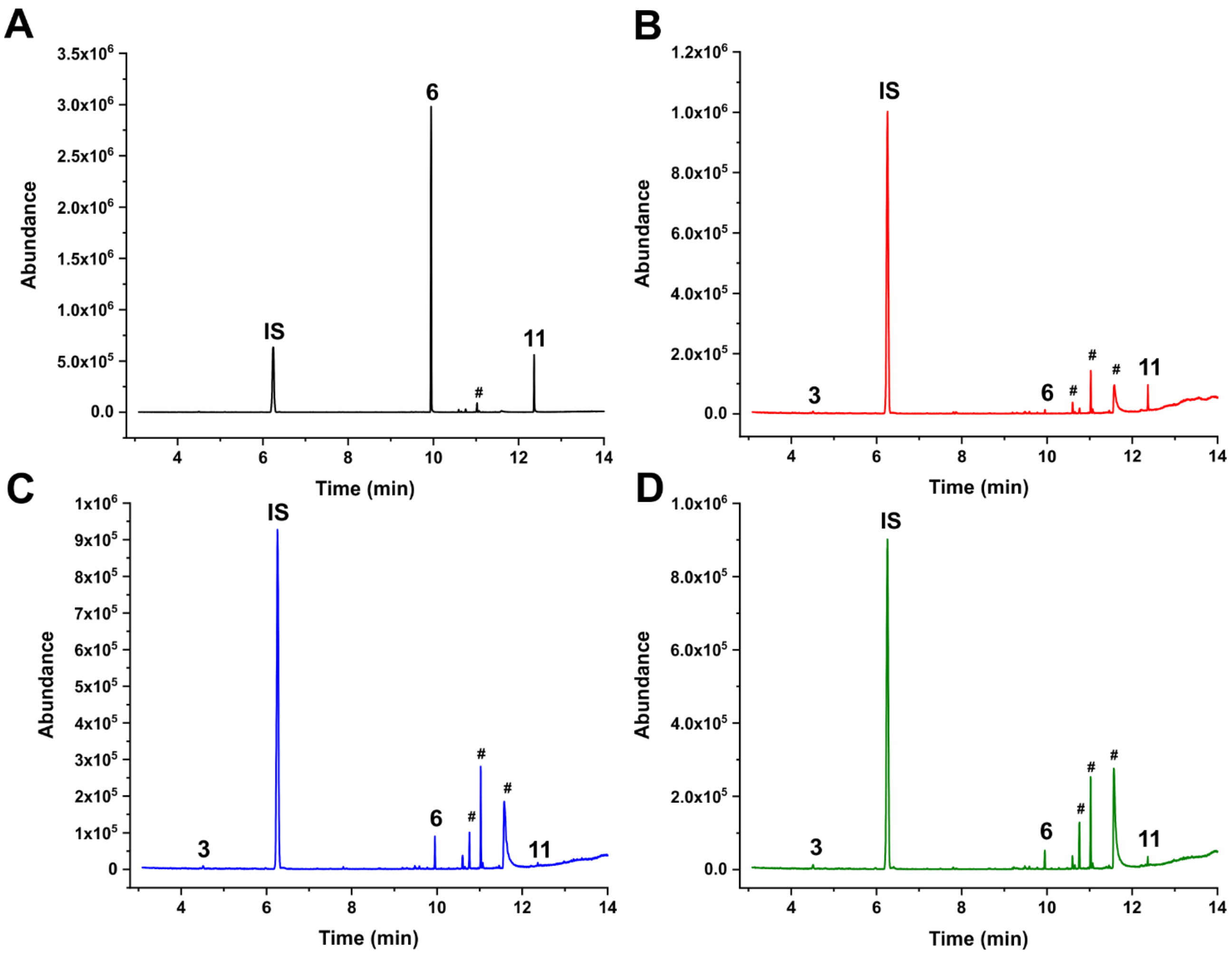

Figure S10: GCMS analysis of terpenoid production strains containing bLinS-F295 mutants. Representative total ion count chromatograms of wild-type bLinS (**A**), bLS-F295A (**B**), bLS-F295W (**C**), and bLS-F295Y (**D**) from *Streptomyces clavuligerus*. Peaks are indicated and labelled as in Figure S1.

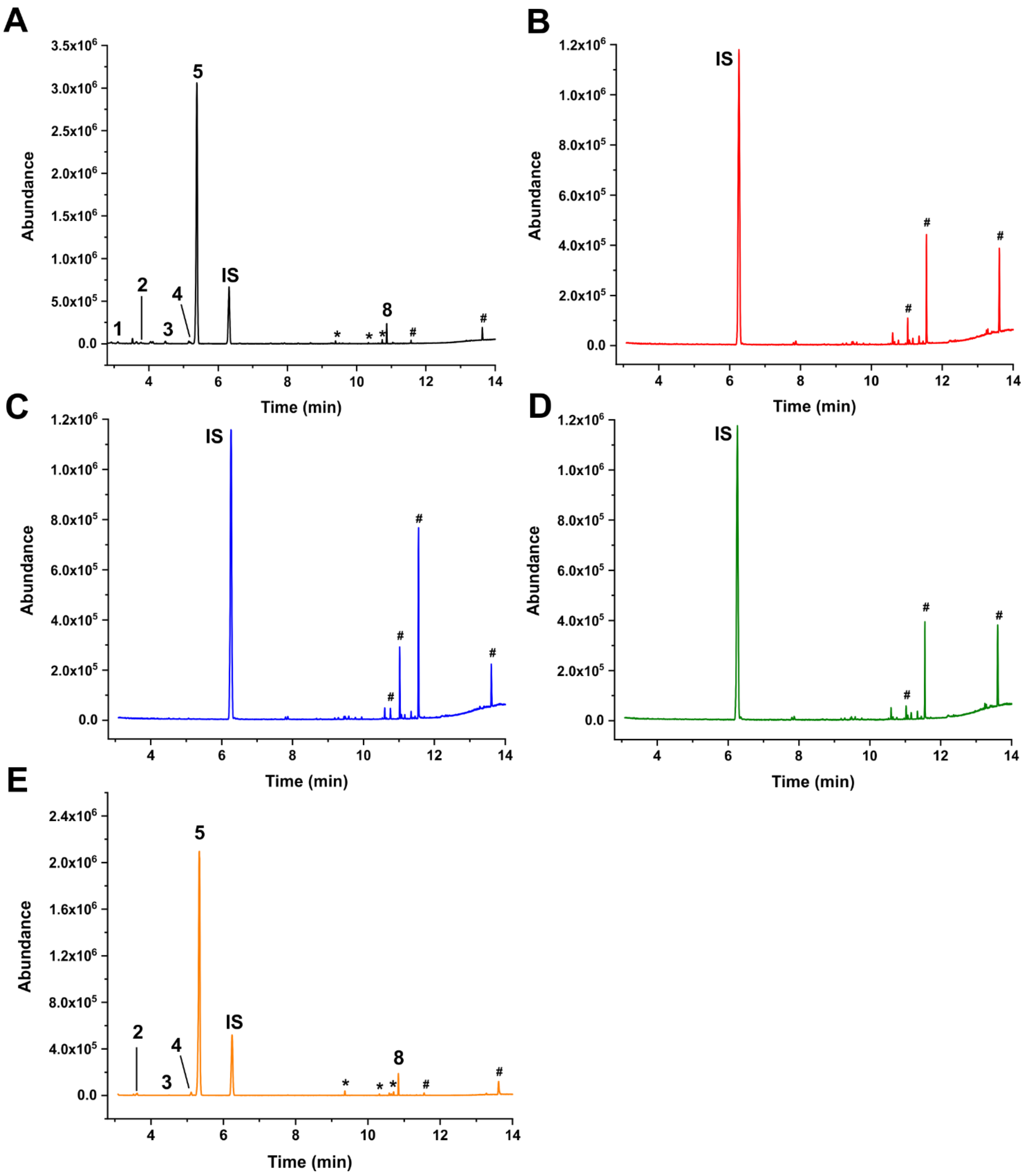

Figure S11: GCMS analysis of terpenoid production strains containing bCinS-A301 mutants. Representative total ion count chromatograms of wild-type bCinS (**A**), bCS-A301F (**B**), bCS-A301L (**C**), bCS-A301V (**D**), and bCS-A301G (**E**) from *Streptomyces clavuligerus*. Peaks are indicated and labelled as in Figure S1.

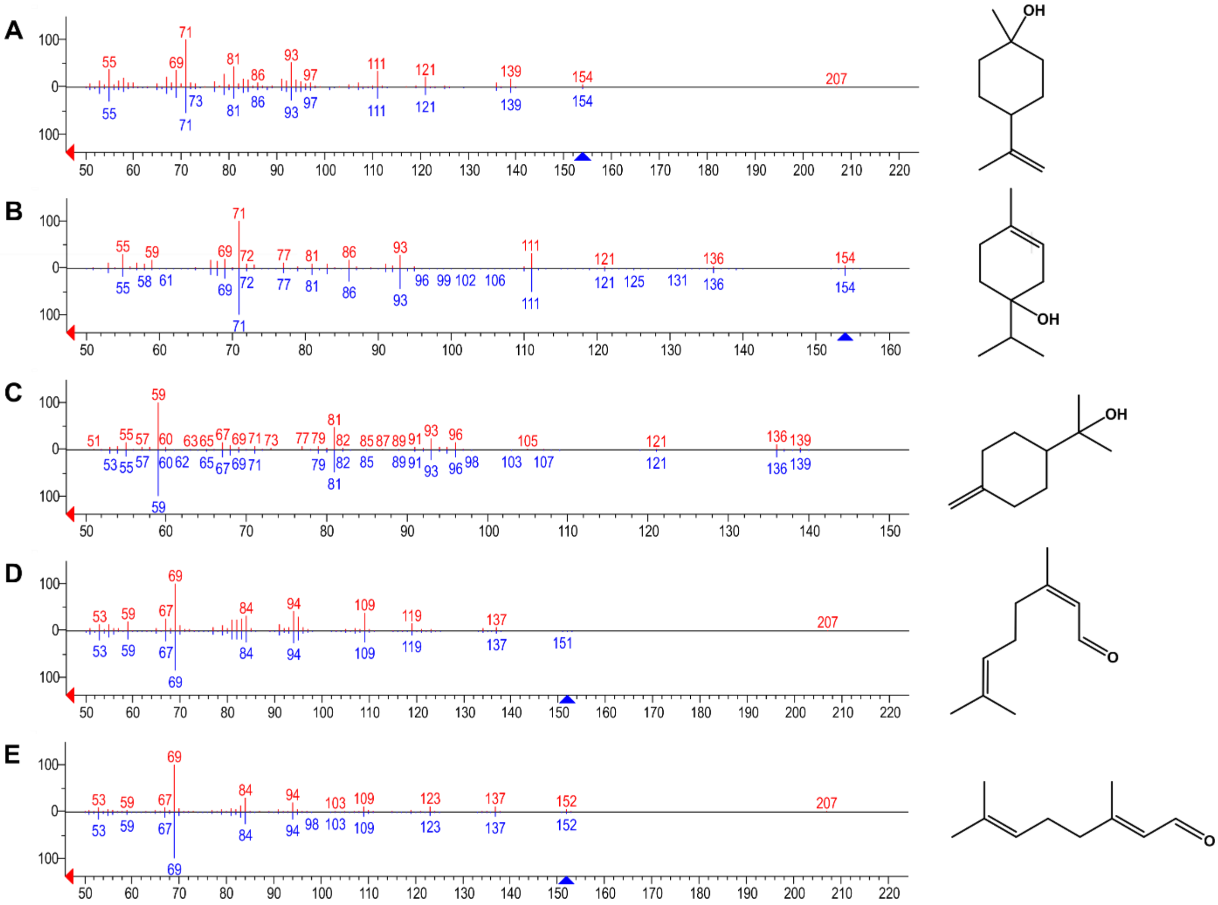

Figure S12: Identification of monoterpenoid products in the absence of authentic standards. Monoterpenoid product MS spectra compared with their closest MS spectra in the NIST database. The y-axes show the relative intensities of each ion and the x-axes the corresponding m/z value. Recorded spectra are in red and spectra obtained from the NIST database are in blue. **A)** β-terpineol (rt = 9.39 min), **B)** 4-terpineol (rt = 10.34 min), **C)** δ-terpineol (rt = 10.74 min), **D)** neral (rt = 10.77 min), **E)** geranial (rt = 10.03 min).

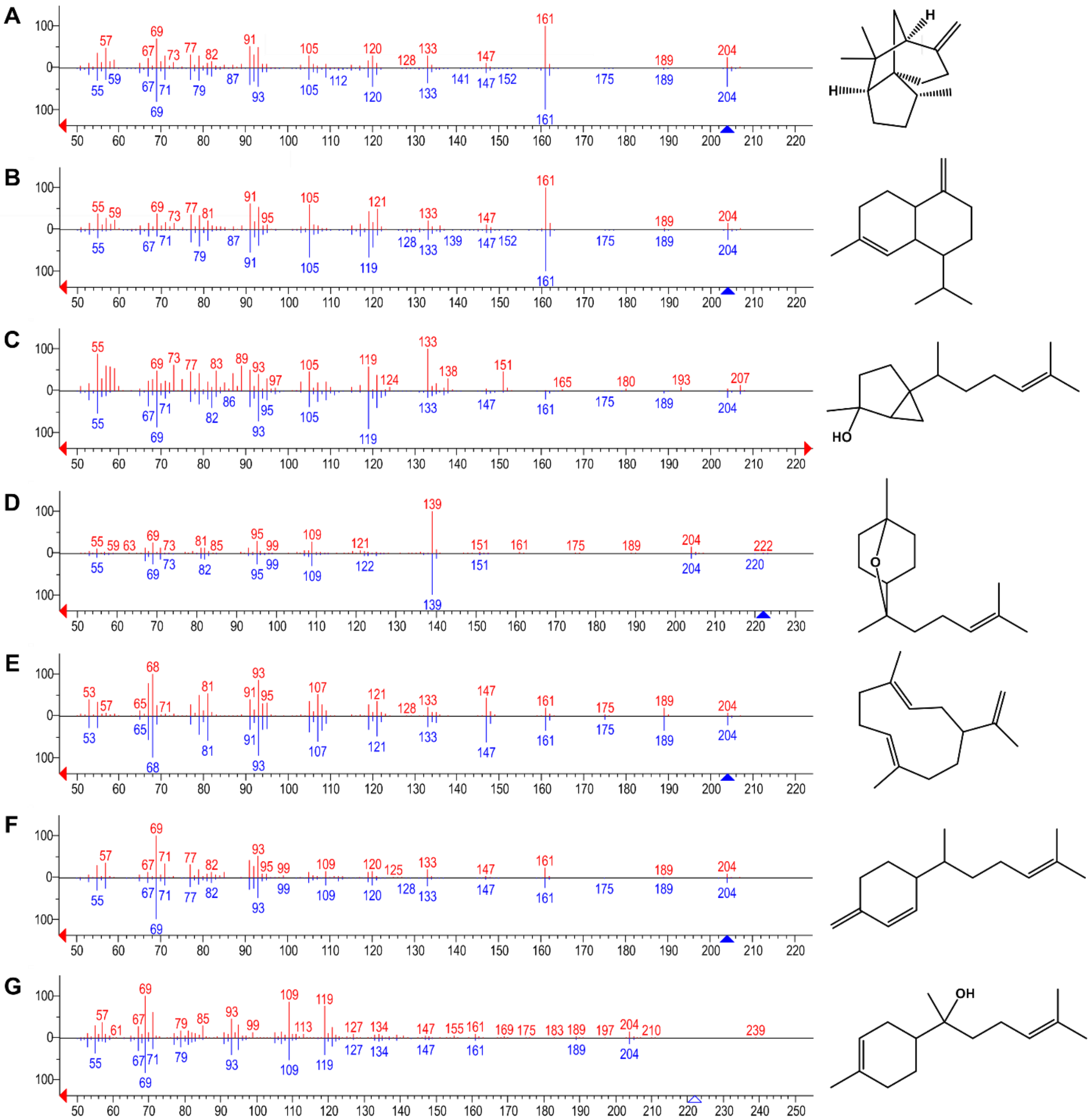

Figure S13: Identification of sesquiterpenoids in the absence of authentic standards. Sesquiterpenoid product MS spectra compared with their closest MS spectra in the NIST database. The y-axes show the relative intensities of each ion and the x-axes the corresponding m/z value. **A**) β-cedrene (rt = 10.26 min), **B**) γ-amorphene (rt = 10.78 min), **C**) sesquisabinene hydrate (rt = 11.09 min), **D**) Sesquicineole (rt = 11.12), **E**) germacrene A (rt = 11.18 min), **F**) β-sesquiphellandrene (rt = 11.23 min), **G**) α-bisabolol (rt = 13.10 min).

Table S3: Retention times and indices for all terpenoid compounds detected in this study. The retention times were obtained using the GC-MS method described in the experimental section using a DB-WAX (high-polarity, polyethylene glycol) column. The Retention Indices are obtained from published sources.

Compound	Authentic Standard	Retention time (min)	RI (PEG)[a]	Reference
Camphene	Yes	3.11	1068.5	[26]
β-pinene	Yes	3.65	1110.0	[26]
Myrcene	Yes	4.54	1160.9	[26]
Limonene	Yes	5.16	1198.2	[26]
1,8-cineole	Yes	5.39	1211.1	[26]
β-terpineol	No	9.39	1639.0	[26]
Linalool	Yes	9.98	1543.3	[26]
β-cedrene	No	10.26	1611.1	[26]
Terpen-4-ol	No	10.34	1601.2	[26]
Neral	No	10.68	1678.5	[26]
δ-terpineol	No	10.74	1679.3	[26]
γ-amorphene	No	10.78	1710.0	[27]
α-terpineol	Yes	10.89	1694.0	[26]
Geranial	No	10.90	1725.0	[26]
Sesquisabinene hydrate	No	11.09	2081.5	[26]
Citronellol	No	11.10	1763.9	[26]
Sesquicineole	No	11.12	1760.0	[28]
Germacrene A	No	11.18	1747.4	[26]
β-sesquiphellandrene	No	11.23	1771.4	[26]
Nerol	Yes	11.30	1794.6	[26]
Geraniol	Yes	11.56	1839.3	[26]
Trans-nerolidol	Yes	12.34	2036.3	[26]
α-bisabolol	No	13.10	2213.6	[26]
Farnesol	Yes	13.61	2366.0	[26]

[a] RI – retention index, PEG – polyethylene glycol

Table S4: Product profiles and titres obtained for wt-bCinS, bLinS and all variants tested in this study. Product profiles and monoterpenoid titres (mg L_{org}^{-1}) were determined from two-phase cultures with a nonane overlay for each *E. coli* strain containing the MVA pathway and a bCinS or bLinS variant. Averages of 3-8 biological replicates and the corresponding standard deviations are shown. ND = not detected

Variant	camphene	β-pinene	β-myrcene	limonene	1,8-cineole	linalool	α-terpineol	geranoids[1]	other (mono)	farnesol[1]	other (sesqui)	Reference
**wt-bCinS and variants**												
wt-bCinS	2.8 ± 1.3	3.5 ± 1.5	0.6 ± 0.6	2.6 ± 1.3	514.5 ± 211.2	ND	11.6 ± 4.9	2.7 ± 2.1	5.1 ± 1.8[2]	18.9 ± 5.0	ND	[2, 14a]
bCS-F74A	ND	ND	2.0 ± 0.2	2.3 ± 0.1	75.9 ± 1.8	23.1 ± 0.4	26.4 ± 0.5	40.9 ± 2.0	9.5 ± 0.2[2]	1.9 ± 0.2	178.5 ± 4.9[3]	This study
bCS-F74V	1.0 ± 0.1	1.2 ± 0.2	0.6 ± 0.2	1.4 ± 0.2	97.8 ± 17.4	0.1 ± 0.2	10.9 ± 1.9	34.8 ± 4.4	0.9 ± 0.6[2]	5.2 ± 1.3	27.7 ± 10.1[4]	This study
bCS-F74L	1.5 ± 1.3	0.9 ± 0.8	0.4 ± 0.5	1.1 ± 1.1	161.6 ± 119.7	ND	3.7 ± 2.7	8.2 ± 7.1	4.1 ± 3.2[2]	9.0 ± 7.0	ND	This study
bCS-F77A	ND	ND	ND	ND	ND	0.3 ± 0.2	ND	42.0 ± 26.1	ND	2.7 ± 0.6	ND	This study
bCS-F77L	ND	ND	ND	ND	ND	0.2 ± 0.3	ND	20.7 ± 10.0	ND	0.7 ± 0.1	ND	This study
bCS-F77Y	ND	0.3 ± 0.4	ND	ND	2.2 ± 1.6	0.5 ± 0.3	0.2 ± 0.1	52.7 ± 35.1	ND	3.2 ± 3.1	ND	This study
bCS-F77W	ND	ND	0.4 ± 0.5	0.3 ± 08	27.4 ± 24.9	1.5 ± 1.6	1.5 ± 1.6	106.2 ± 95.6	ND	16.1 ± 14.8	ND	This study
bCS-F77H	ND	ND	ND	0.2 ± 0.5	ND	0.2 ± 0.4	0.6 ± 1.0	73.5 ± 67.7	ND	17.0 ± 1.9	ND	This study
bCS-F78A	ND	ND	ND	ND	ND	0.3 ± 0.4	ND	41.4 ± 24.8	ND	2.3 ± 4.6	ND	This study
bCS-F78V	ND	ND	0.1 ± 0.2	ND	5.1 ± 5.0	0.1 ± 0.2	0.1 ± 0.2	21.1 ± 16.4	ND	4.4 ± 2.0	ND	This study
bCS-F78L	0.7 ± 0.1	1.0 ± 0.3	0.2 ± 0.4	1.2 ± 0.4	111.2 ± 32.2	0.2 ± 0.4	17.1 ± 5.1	36.8 ± 8.3	4.0 ± 0.9[2]	9.7 ± 1.2	ND	This study
bCS-F179A	3.4 ± 1.4	0.6 ± 0.1	1.0 ± 0.4	3.0 ± 1.2	231.2 ± 91.7	ND	10.5 ± 4.1	4.5 ± 2.9	4.0 ± 1.8[2]	6.9 ± 6.3	19.0 ± 8.0[5]	This study
bCS-F179V	0.8 ± 1.0	0.4 ± 0.4	0.1 ± 0.2	0.6 ± 05	98.3 ± 62.7	0.1 ± 0.2	3.6 ± 2.3	22.5 ± 11.9	3.0 ± 1.7[2]	2.7 ± 3.7	ND	This study
bCS-F179L	0.3 ± 0.5	ND	0.1 ± 0.2	0.1 ± 0.3	23.0 ± 16.6	0.1 ± 0.1	1.2 ± 0.9	27.9 ± 9.8	0.1 ± 0.1	5.3 ± 4.0	ND	This study
bCS-A301F	ND	ND	ND	ND	ND	0.1 ± 0.1	ND	15.1 ± 12.3	ND	5.3 ± 4.9	ND	This study
bCS-A301L	ND	ND	0.1 ± 0.2	ND	ND	0.2 ± 0.1	ND	22.0 ± 17.2	ND	3.5 ± 2.8	ND	This study
bCS-A301V	ND	ND	0.1 ± 0.1	0.1 ± 0.1	ND	0.1 ± 0.1	ND	17.7 ± 7.1	ND	6.3 ± 4.5	ND	This study
bCS-A301G	ND	5.2 ± 2.3	0.4 ± 0.2	4.1 ± 2.0	484.6 ± 186.4	0.1 ± 0.1	10.9 ± 4.4	6.2 ± 7.4	5.7 ± 2.8[2]	11.5 ± 3.3	ND	This study
**wt-bLinS and variants**												
wt-bLinS	ND	ND	ND	ND	ND	363.3 ± 57.9	ND	36.7 ± 0.8	ND	31.5 ± 3.7	159.1 ± 7.9[6]	[2]
bLS-F76A	ND	ND	0.5 ± 0.4	ND	ND	0.7 ± 0.3	ND	43.5 ± 27.7	ND	2.8 ± 2.9	ND	This study
bLS-F295A	ND	ND	0.1 ± 0.2	ND	ND	0.3 ± 0.2	ND	29.1 ± 15.8	ND	3.6 ± 3.4	2.6 ± 4.6[6]	This study
bLS-F295W	ND	ND	0.4 ± 0.5	ND	ND	2.9 ± 2.5	ND	59.6 ± 55.7	ND	ND	0.3 ± 0.1[6]	This study
bLS-F295Y	ND	ND	0.6 ± 0.3	ND	ND	1.2 ± 1.3	ND	60.0 ± 51.2	ND	ND	0.5 ± 0.4[6]	This study

[1]Geraniol and derivatives (geranoids) as well as farnesol production is most likely the result of endogenous *E. coli* activity, and includes the compounds nerol, neral, and geranial [1, 25a]

[2]Other monoterpenoids detected for wt-bCinS, bCS-F74A, bCS-F74L, bCS-F78L, bCS-F179A, bCS-F179V and bCS-A301G include: β-terpineol, 4-terpineol, and δ-terpineol

[3]Sesquiterpenoids detected for bCS-F74A include: bisabolene (2.4 ± 0.1 mg L_{org}^{-1}), sesquicineole (126.4 ± 3.3 mg L_{org}^{-1}), β-sesquiphellandrene (5.2 ± 0.2 mg L_{org}^{-1}), and α-bisabolol (44.5 ± 1.4 mg L_{org}^{-1})

[4]Sesquiterpenoids detected for bCS-F74V include: sesquicineole (13.0 ± 4.4 mg L_{org}^{-1}), β-sesquiphellandrene (1.7 ± 1.3 mg L_{org}^{-1}), and α-bisabolol (13.0 ± 4.4 mg L_{org}^{-1})

[5]Sesquiterpenoids detected for bCS-F179A include: β-cedrene (1.7 ± 0.7 mg L_{org}^{-1}), germacrene A (11.1 ± 4.7 mg L_{org}^{-1}), γ-amorphene (2.2 ± 1.0 mg L_{org}^{-1}), sesquisabinene hydrate, and β-sesquiphellandrene (≤1.0 mg L_{org}^{-1} each)

[6]Sesquiterpenoid detected for wt-bLinS, bLS-F295A, bLS-F295W and bLS-F295Y: *trans*-nerolidol

REFERENCES

[1] N. G. H. Leferink, A. J. Jervis, Z. Zebec, H. S. Toogood, S. Hay, E. Takano, N. S. Scrutton, *ChemistrySelect* **2016**, *1*, 1893-1896.

[2] V. Karuppiah, K. E. Ranaghan, N. G. H. Leferink, L. O. Johannissen, M. Shanmugam, A. Ni Cheallaigh, N. Bennett, L. Kearsey, E. Takano, J. Gardiner, M. W. Van der Kamp, S. Hay, A. J. Mulholland, D. Leys, N. S. Scrutton, *ACS Catal.* **2017**, *7*, 6268-6282.

[3] C. A. Ferraz, N. G. H. Leferink, I. Kosov, N. Scrutton, *Chembiochem* **2021**, *22*, 2325-2334.

[4] a) M. H. M. Olsson, C. R. Søndergaard, M. Rostkowski, J. H. Jensen, *J. Chem. Theory Comput.* **2011**, *7*, 525-537; b) C. R. Søndergaard, M. H. M. Olsson, M. Rostkowski, J. H. Jensen, *J. Chem. Theory Comput.* **2011**, *7*, 2284-2295.

[5] a) H. M. Senn, W. Thiel, in *Atomistic Approaches in Modern Biology: From Quantum Chemistry to Molecular Simulations* (Ed.: M. Reiher), Springer Berlin Heidelberg, Berlin, Heidelberg, **2007**, pp. 173-290; b) M. W. van der Kamp, A. J. Mulholland, *Biochemistry* **2013**, *52*, 2708-2728.

[6] Y. Zhao, D. G. Truhlar, *Theor. Chem. Account.* **2008**, *120*, 215-241.

[7] a) R. B. Best, X. Zhu, J. Shim, P. E. M. Lopes, J. Mittal, M. Feig, A. D. MacKerell, *J. Chem. Theory Comput.* **2012**, *8*, 3257-3273; b) C. Lee, W. Yang, R. G. Parr, *Phys. Rev. B* **1988**, *37*, 785-789.

[8] a) S. Metz, J. Kästner, A. A. Sokol, T. W. Keal, P. Sherwood, *WIREs Comput. Mol. Sci.* **2014**, *4*, 101-110; b) P. Sherwood, A. H. de Vries, M. F. Guest, G. Schreckenbach, C. R. A. Catlow, S. A. French, A. A. Sokol, S. T. Bromley, W. Thiel, A. J. Turner, S. Billeter, F. Terstegen, S. Thiel, J. Kendrick, S. C. Rogers, J. Casci, M. Watson, F. King, E. Karlsen, M. Sjøvoll, A. Fahmi, A. Schäfer, C. Lennartz, *J. Mol. Struct. THEOCHEM* **2003**, *632*, 1-28.

[9] M. J. Frisch, G. Trucks, H. Schlegel, G. Scuseria, M. Robb, J. Cheeseman, G. Scalmani, V. Barone, B. Mennucci, G. Petersson, *Gaussian 09 D. 01*, Wallingford CT **2016**.

[10] W. Smith, T. R. Forester, *J. Mol. Graphics* **1996**, *14*, 136-141.

[11] a) S. R. Billeter, A. J. Turner, W. Thiel, *Phys. Chem. Chem. Phys.* **2000**, *2*, 2177-2186; b) J. Kästner, J. M. Carr, T. W. Keal, W. Thiel, A. Wander, P. Sherwood, *J. Phys. Chem. A* **2009**, *113*, 11856-11865.

[12] a) D. Bakowies, W. Thiel, *J. Phys. Chem.* **1996**, *100*, 10580-10594; b) A. H. de Vries, P. Sherwood, S. J. Collins, A. M. Rigby, M. Rigutto, G. J. Kramer, *J. Phys. Chem. B* **1999**, *103*, 6133-6141.

[13] a) A. Schäfer, C. Huber, R. Ahlrichs, *J. Chem. Phys.* **1994**, *100*, 5829-5835; b) S. F. Boys, F. Bernardi, *Mol. Phys.* **1970**, *19*, 553-566; c) S. Simon, M. Duran, J. J. Dannenberg, *J. Chem. Phys.* **1996**, *105*, 11024-11031.

[14] a) N. G. H. Leferink, K. E. Ranaghan, J. Battye, L. O. Johannissen, S. Hay, M. W. van der Kamp, A. J. Mulholland, N. S. Scrutton, *Chembiochem* **2020**, *21*, 985-990; b) P. L. Srivastava, A. M. Escorcia, F. Huynh, D. J. Miller, R. K. Allemann, M. W. van der Kamp, *ACS Catal.* **2021**, *11*, 1033-1041.

[15] W. Humphrey, A. Dalke, K. Schulten, *J. Mol. Graphics* **1996**, *14*, 33-38.

[16] a) D. A. Case, R. M. Betz, D. S. Cerutti, T. E. Cheatham, III, T. A. Darden, R. E. Duke, T. J. Giese, H. Gohlke, A. W. Goetz, N. Homeyer, S. Izadi, P. Janowski, J. Kaus, A. Kovalenko, T. S. Lee, S. LeGrand, P. Li, C. Lin, T. Luchko, R. Luo, B. Madej, D. Mermelstein, K. M. Merz, G. Monard, H. Nguyen, H. T. Nguyen, I. Omelyan, A. Onufriev, D. R. Roe, A. Roitberg, C. Sagui, C. L. Simmerling, W. M. Botello-Smith, J. Swails, R. C. Walker, J. Wang, R. M. Wolf, X. Wu, L. Xiao, P. A. Kollman, *AMBER 2016*, University of California, San Francisco, **2016**; b) M. F. Crowley, M. J. Williamson, R. C. Walker, *Int. J. Quantum Chem.* **2009**, *109*, 3767-3772; c) A. W. Götz, M. J. Williamson, D. Xu, D. Poole, S. Le Grand, R. C. Walker, *J. Chem. Theory Comput.* **2012**, *8*, 1542-1555.

[17] M. W. van der Kamp, J. Sirirak, J. Zurek, R. K. Allemann, A. J. Mulholland, *Biochemistry* **2013**, *52*, 8094-8105.

[18] O. Allnér, L. Nilsson, A. Villa, *J. Chem. Theory Comput.* **2012**, *8*, 1493-1502.

[19] N. G. H. Leferink, K. E. Ranaghan, V. Karuppiah, A. Currin, M. W. van der Kamp, A. J. Mulholland, N. S. Scrutton, *ACS Catal.* **2018**, *8*, 3780-3791.

[20] J.-P. Ryckaert, G. Ciccotti, H. J. C. Berendsen, *J. Comput. Phys.* **1977**, *23*, 327-341.

[21] P. Baer, P. Rabe, K. Fischer, C. A. Citron, T. A. Klapschinski, M. Groll, J. S. Dickschat, *Angew. Chem. Int. Ed. Engl.* **2014**, *53*, 7652-7656.

[22] J. A. Aaron, X. Lin, D. E. Cane, D. W. Christianson, *Biochemistry* **2010**, *49*, 1787-1797.

[23] P. Baer, P. Rabe, C. A. Citron, C. C. de Oliveira Mann, N. Kaufmann, M. Groll, J. S. Dickschat, *Chembiochem* **2014**, *15*, 213-216.

[24] C. A. Lesburg, G. Zhai, D. E. Cane, D. W. Christianson, *Science* **1997**, *277*, 1820-1824.

[25] a) W. Liu, R. Zhang, N. Tian, X. Xu, Y. Cao, M. Xian, H. Liu, *Bioengineered* **2015**, *6*, 288-293; b) J. Zhou, C. Wang, S.-H. Yoon, H.-J. Jang, E.-S. Choi, S.-W. Kim, *J. Biotechnol.* **2014**, *169*, 42-50; c) T. Wang, J. Guo, Y. Liu, Z. Xue, C. Zhang, X.-H. Xing, *Appl. Microbiol. Biotechnol.* **2018**, *102*, 9771-9780.

[26] V. I. Babushok, P. J. Linstrom, I. G. Zenkevich, *J. Phys. Chem. Ref. Data* **2011**, *40*, 043101.

[27] J. B. Boti, P. A. Yao, G. Koukoua, T. Y. N'Guessan, J. Casanova, *Flavour Fragr. J.* **2006**, *21*, 166-170.

[28] P. Weyerstahl, H. Marschall, K. Thefeld, G. C. Subba, *Flavour Fragr. J.* **1998**, *13*, 377-388.
